# Inhibition of Drp1- Fis1 interaction alleviates aberrant mitochondrial fragmentation and acute kidney injury

**DOI:** 10.1186/s11658-024-00553-1

**Published:** 2024-03-04

**Authors:** Zhixia Song, Yao Xia, Lang Shi, Hongchu Zha, Jing Huang, Xiaohong Xiang, Huiming Li, Hua Huang, Ruchi Yue, Hongtao Wang, Jiefu Zhu

**Affiliations:** 1 Department of Nephrology, Center People’s Hospital of Yichang, The First Clinical Medical College of Three Gorges University, Yichang, 443000 Hubei China; 2 Kidney Disease Research Institute of Three Gorges University, Yichang, 443000 Hubei China; 3 Department of Nephrology, Renmin Hospital of Wuhan University, Wuhan, 430060 China; 4 Department of Critical Care Medicine, The Second Xiangya Hospital, Central South University, Changsha, 410000 China; 5 Department of Urology, Renmin Hospital of Wuhan University, Wuhan, 430060 China; 6 Department of Organ Transplantation, Renmin Hospital of Wuhan University, Wuhan, 430060 China

**Keywords:** Acute kidney injury, Ischemia reperfusion injury, Mitochondria, Drp1, Fis1

## Abstract

**Background:**

Acute kidney injury (AKI) is a common clinical disorder with complex etiology and poor prognosis, and currently lacks specific and effective treatment options. Mitochondrial dynamics dysfunction is a prominent feature in AKI, and modulation of mitochondrial morphology may serve as a potential therapeutic approach for AKI.

**Methods:**

We induced ischemia–reperfusion injury (IRI) in mice (bilateral) and Bama pigs (unilateral) by occluding the renal arteries. ATP depletion and recovery (ATP-DR) was performed on proximal renal tubular cells to simulate in vitro IRI. Renal function was evaluated using creatinine and urea nitrogen levels, while renal structural damage was assessed through histopathological staining. The role of Drp1 was investigated using immunoblotting, immunohistochemistry, immunofluorescence, and immunoprecipitation techniques. Mitochondrial morphology was evaluated using confocal microscopy.

**Results:**

Renal IRI induced significant mitochondrial fragmentation, accompanied by Dynamin-related protein 1 (Drp1) translocation to the mitochondria and Drp1 phosphorylation at Ser616 in the early stages (30 min after reperfusion), when there was no apparent structural damage to the kidney. The use of the Drp1 inhibitor P110 significantly improved kidney function and structural damage. P110 reduced Drp1 mitochondrial translocation, disrupted the interaction between Drp1 and Fis1, without affecting the binding of Drp1 to other mitochondrial receptors such as MFF and Mid51. High-dose administration had no apparent toxic side effects. Furthermore, ATP-DR induced mitochondrial fission in renal tubular cells, accompanied by a decrease in mitochondrial membrane potential and an increase in the translocation of the pro-apoptotic protein Bax. This process facilitated the release of dsDNA, triggering the activation of the cGAS-STING pathway and promoting inflammation. P110 attenuated mitochondrial fission, suppressed Bax mitochondrial translocation, prevented dsDNA release, and reduced the activation of the cGAS-STING pathway. Furthermore, these protective effects of P110 were also observed renal IRI model in the Bama pig and folic acid-induced nephropathy in mice.

**Conclusions:**

Dysfunction of mitochondrial dynamics mediated by Drp1 contributes to renal IRI. The specific inhibitor of Drp1, P110, demonstrated protective effects in both in vivo and in vitro models of AKI.

**Supplementary Information:**

The online version contains supplementary material available at 10.1186/s11658-024-00553-1.

## Introduction

AKI refers to a rapid and severe decline in renal function, characterized by a rapid increase in serum creatinine levels and a decrease in urine output [1]. AKI commonly occurs in conditions such as infection, nephrotoxic drugs, and hypovolemic shock. The incidence of AKI in hospitalized patients is approximately 10–15%, but it is reported to be even higher in intensive care units, often exceeding 50%, with a high mortality rate [2]. Even among survivors, there is a significant increase in the proportion of individuals progressing to chronic kidney disease (CKD) or end-stage renal disease (ESRD) compared to the general population [3, 4]. Currently, there are no specific therapeutic interventions for AKI, and supportive care remains the mainstay of treatment. Therefore, there is an urgent need to elucidate the pathogenesis of acute kidney injury and identify specific treatments.

Mitochondria play a critical role in cellular energy metabolism and maintaining redox balance [5, 6]. The kidney, being an organ with a high energy demand, contains a significant number of mitochondria, especially in renal tubules [7]. It has been well established by our studies and others that mitochondrial damage is a key pathogenic mechanism underlying acute kidney injury, and perturbations in mitochondrial dynamics contribute to the development of this condition [8–11]. Mitochondrial dynamics are finely regulated by a balance between mitochondrial fusion proteins and mitochondrial fission proteins. Among these proteins, Dynamin-related protein 1 (Drp1) plays a crucial role in the process of mitochondrial fission. Drp1 is primarily located in the cytoplasm and, upon activation, translocates to the mitochondrial outer membrane by interacting with receptor proteins such as mitochondrial fission factor (MFF), Mitochondrial Elongation Factor 1(Mid51), and Mitochondrial Fission 1(Fis1), thus carrying out its function [12, 13]. Aberrant Drp1 activation leads to mitochondrial dysfunction, exacerbating ischemic injury [14], neurodegeneration diseases [15], and sepsis [16, 17].

Despite Drp1 being an attractive pharmacological target, the activity of Drp1 is essential for maintaining many physiological functions, and the knockout of Drp1 exhibits cytotoxic effects [18, 19]. Short-term use of targeted inhibitors is a preferable option. Mdivi-1 was initially identified as a yeast mitochondrial fission inhibitor through chemical library screening [20] and Mdivi-1 is widely utilized as a Drp1 inhibitor [21–23]. However recent research indicates that Mdivi-1 reversibly inhibits mitochondrial complex I without compromising Drp1 GTPase activity or prolonging mitochondrial elongation [24–26]. P110 is a selective peptide inhibitor of the GTPase activity of Drp1, designed based on the interacting regions of Drp1 and Fis1 [15, 17, 27]. Given Drp1's pivotal role in mitochondrial fission under normal conditions, determining the impact of Drp1 inhibition on cellular bioenergetics in the short or long term is of paramount importance.

In this study, we aimed to elucidate the mechanisms of mitochondrial dynamics and the role of the associated protein Drp1, as well as the effects of P110 in the context of AKI. Our findings revealed that P110, through the selective inhibition of Drp1 activity, exerts strong and significant protective effects in both mice AKI and Bama pig IRI models. These findings suggest that P110 has the potential for precise regulation mitochondrial dynamics without requiring genetic editing, demonstrating its effectiveness in alleviating IRI and highlighting its promise as an innovative treatment for AKI.

## Materials and methods

### Animals models of AKI and treatment

Male C57BL/6 J mice were bought from Three Gorges University's Experimental Animal Center. The bilateral renal pedicle was clamped for 28 min with a microaneurysm clamp, as indicated previously [28]. In the sham surgery, the renal pedicle was exposed without being clamped. Reperfusion was then conducted at various time intervals of 0.5 h, 2 h, 6 h, and 24 h. P110 peptides and fragment of the HIV transactivator protein (TAT) (dissolved in saline) were synthesized by Selleck and Genscript company. P110 and TAT was intraperitoneally injected into mice at various time points and dosages based on our prior research, whereas control animals received the same quantity of saline daily to mimic a similar therapy.

Eight Bamach miniature pigs weighing 12.65 ± 1.12 kg each were isolated (provided by Wuhan Wuguan Medical Technology Co., Ltd.) and allowed to acclimate for 2 weeks. The pigs were randomly divided into two groups, TAT group and P110 group. At 24 h and 1 h before surgery, 0.4 mg/kg of P110 and TAT were intravenously injected into the respective groups. The animals were fasted for 12 h before the surgery. Prior to the start of the surgery, anesthesia was induced using Zoletil 50 (Virbac, France) through intramuscular injection. Each pig underwent endotracheal intubation and was maintained under anesthesia with 2% isoflurane. They were connected to a ventilator and a sophisticated electrocardiogram monitoring device. Additionally, a venous catheter was placed in the ear. A left-sided dorsal incision was made, and the left renal hilum was exposed through the retroperitoneum. The renal artery of the left kidney was clamped for 120 min. Subsequently, a right-sided dorsal incision was made, and the right renal hilum was exposed through the retroperitoneum for right nephrectomy, obtaining the right kidney as the sham group. At 24 and 48 h post-surgery, venous blood samples were collected to assess renal function. After 72 h of reperfusion, the left kidney and serum were collected under anesthesia. Kidney tissues at the cortex-medulla junction were rapidly frozen or preserved in 4% formaldehyde. Euthanasia was performed by intravenous injection of potassium chloride.

Folic acid-induced nephropathy was induced with a single intraperitoneal injection of folic acid (250 mg/kg body weight in 0.3 M sodium bicarbonate solution) in C57/Bl6 male mice. Kidney tissue and serum samples were extracted 48 h after folic acid treatment for kidney function, protein expression and histological analyses.

### Renal histology

For paraffin embedding, fresh kidneys were taken and treated with 4% paraformaldehyde overnight at 4 °C. Hematoxylin and eosin (H&E) and Periodic Acid Schiff (PAS)  staining was applied to 4-mm slices of the paraffin-embedded tissues. Histopathologists scored the degree of kidney injury in a blind manner. According to renal tubular injury estimation, the following histological damage was graded: Damage levels are 0, no damage; 1, < 25; 2, 25–50%; 3, 50–75%; and 4, > 75%.

### Renal function measurement

The central laboratory of the Center People's Hospital of Yichang (Roche Diagnostics GmbH, Penzberg and Mannheim, Germany) analyzed the levels of plasma creatinine and BUN.

### Immunofluorescence and immunohistochemistry analysis

The paraffin sections were deparaffinized and subjected to antigen retrieval by steaming with sodium citrate buffer. mPTCs grown on collagen-coated coverslips were fixed with 4% paraformaldehyde. Primary antibodies for immunofluorescence included Ki67 (Cell Signaling Technology, 9129T), Bax (Abcam, ab182733), DLP1(BD Bioscience, 611112), Fis1 (Proteintech, 10956-1-AP) and dsDNA (Abcam,ab27156). Anti-rabbit or anti-goat or Alexa Fluor 647 conjugated secondary antibodies were used, and the fluorescent signals were visualized under a fluorescence microscope. Primary antibodies for immunohistochemical staining included KIM-1(R&D System, AF1750), PGC1 α (Novusbio, NBPI1-04676SS), COX I (Bioss, bs-3953R), STING(Cell Signaling Technology, 13647S),F4/80(Starter, S0B0227) and NGAL(R&D System, AF1757). The nucleus was counterstained with hematoxylin, dried, and mounted after being counterstained with DAB for color development. Using Image-Pro plus 6.0 software, positive staining regions were quantified.

### Examination of mitochondrial fragmentation in kidney tissues

The kidney tissue was perfused with 1 ml of 10 U/ml heparin, followed by 2 ml fixative containing 100 mM sodium cacodylate, 2 mM calcium chloride, 4 mM magnesium sulfate, 4% paraformaldehyde, and 2.5% glutaraldehyde through the abdominal aorta. From each kidney, a tissue block measuring roughly 1 mm^3^ was taken, along with some renal cortex and outer medulla tissue for standard processing for electron microscopy. To find representative proximal tubules, the tissue block was initially examined at a modest magnification (× 3000). Then, high-magnification (× 15,000) examination of the cells in these tubules was performed to get electron micrographs.

### Analysis of mitochondrial fragmentation

Plasmid of pDsRed2-mito was transfected into mPTC using Lipofecmin 2000 to visualize mitochondria. Using confocal microscopy, mitochondrial morphology was analyzed after cells were treated with 4% paraformaldehyde. For each slide, 150–200 cells from ten randomly chosen places were evaluated to determine the percentage of cells with mitochondrial fragmentation in accordance with the previous research [11, 29, 30].

### Cell culture and treatments

The mouse proximal tubular cells (mPTCs) line was provided by Sciencell Research Laboratories, and all cells were grown with 10% fetal bovine serum + 1% dual antibody + DMEM in an incubator with 5% CO_2_ at 37 °C. A 35 mm culture dish of 1.0 × 10^6^ mPTCs was used, and intervention was carried out when the cell growth density reached 80%–90%. The cells were treated in antimycin 2 μM and oligomycin 2 μM in Krebs–Ringer bicarbonate solution (in mM: 115 NaCl, 1 KH_2_PO_4_, 4 KCl, 1 MgSO_4_, 1.25 CaCl_2_, 25 NaHCO_3_, pH 7.4) with for 3 h to cause ATP depletion, followed by 2 h of recovery in normal culture media, to create the ATP-depletion Recovery (ATP-DR) model [31]. P110 or TAT was added at a 2 μM concentration three hours before to the intervention.

### Isolation of cytosolic and mitochondrial fractions

Cells were divided into cytosolic and mitochondrial fractions using a mitochondria isolation buffer that contained, with some minor modifications from our earlier work, 225 mM mannitol, 75 mM sucrose, 1 mM ethylene glycol tetraacetic acid, 10 mM Tris-hydrochloride, and protein inhibitor cocktail (pH 7.4). In brief, cells were rinsed with ice-cold PBS and suspended in a cold mitochondria isolation buffer.Then the cells were homogenized by passing through a syringe with a 27-gauge needle five times. The homogenates were centrifuged at 800 × *g* for 10 min at 4 °C to remove cell debris and nuclei followed by centrifugation at 15,000 × *g* for 10 min to supernatant the supernatant as cytosolic fraction and the pellet as mitochondrial fraction Fresh kidney tissues were homogenized in a mitochondria isolation buffer containing 0.1% BSA. The homogenates underwent several rounds of centrifugation at 1000 × *g* for 10 min to eliminate cell debris and nuclei, followed by centrifugation at 15,000 × *g* for 15 min to collect the supernatant as the cytosolic fraction and the pellet as the mitochondrial fraction.

### Measurement of mitochondrial membrane potential and ROS

The mitochondrial membrane potential in living cells was measured with the mitochondrial membrane potential sensitive dye JC-1,which was purchased from MCE (CAS No. 3520–43-2). The cells were treated, and 20 uM of JC-1 (dissolved in DMSO) was added. The cells were then cultured in a 5% CO_2_ incubator at 37 °C for 15–20 min. An inverted fluorescence microscope was used to measure the fluorescence of the samples.

MitoSOX (Thermo Fisher) was employed for the visualization of mitochondrial reactive oxygen species (ROS). Following the instructions, 5 μM MitoSOX dye (dissolved in DMSO) was applied to the treated cells. The cells were then incubated at 37 °C for 10 min in a 5% CO_2_ incubator and subsequently washed three times with gentle PBS. Finally, fluorescence images were acquired and examined using an inverted fluorescence microscope.

### Co-immunoprecipitation

Thermo Scientific's Pierce's Classic IP Kit, item number 88828, was employed. The pre-treated cells were first lysed with lysate, the antibodies were then incubated with magnetic beads for 30 to 60 min, the lysate was then incubated with the coupled magnetic beads for 2 h or overnight at 4 °C, and a single antibody was added. Finally, the magnetic beads were eluted with eluent to obtain the target protein. Electrophoresis using SDS-PAGE was used to evaluate the related proteins.

### RNA extraction and quantitative real-time PCR

The kidney tissue or cell RNA was extracted with RNeasy Plus Extraction Kit (TranGen Biotech) and mRNA was obtained by reverse transcription using PrimeScript RT Master Mix (Vazyme). The StepOnePlus Real-Time PCR System (Applied Biosystems) was used to carry out the quantitative real-time PCR using Fast SYBR Green Master Mix (Vazyme) and the cDNA as the template. The internal check for the processes employed glyceraldehyde 3-phosphate dehydrogenase. The comparative cycle threshold (2-ΔΔCt) approach was used to calculate the relative levels of gene expression. Additional file 7: Table S1 contains a list of primer sequences.

### Immunoblotting analysis

The protein content of kidney lysates or cultured cells was assessed using the Pierce bicinchoninic acid reagent from Thermo Fisher Scientific. A similar number of protein samples were loaded for electrophoresis on an SDS–polyacrylamide gel and electroblotting onto a PVDF membrane. The blots were incubated with the main antibody at 4 °C overnight after blocking in 5% non-fat milk or bovine serum albumin, and then with the HRP-conjugated secondary antibody. Following an incubation period with Super Signal West Pico Chemiluminescent Substrate from Thermo Fisher Scientific, the blots were scanned using either a KwikQuant Imager from Kindle Bioscience or a MyECL Imager from Thermo Fisher Scientific to capture the signals. The primary antibodies used for immunoblotting were as follows: p-Drp1(Ser616) (Cell Signaling Technology, 3455S), Cleaved-Caspase-3(Cell Signaling Technology, 9661T), Sirt3(Cell Signaling Technology, 2627 s), cGAS (Cell Signaling Technology, 31659), STING (Cell Signaling Technology, 13647) NF-kappaBp65(Cell Signaling Technology, 8242 T), p-NF-kappaBp65(s536) (Cell Signaling Technology, 3033T), p-IRF3 (Cell Signaling Technology, 4947), TBK1/NAK(Cell Signaling Technology, 3504), Phospho-TBK1/NAK(Ser172)(Cell Signaling Technology, 5483), STING(Abcam, ab288157), DLP1(BD Bioscience, 611116), COX IV(Abcam, ab16056), Bax(Abcam, ab182733), PGC1α(ABclonal, A20995), TFAM(ABclonal, A3173), IRF3((ABclonal, A19717), phospho-IRF3-S386(ABclonal, AP0995),MCP-1(Santa Cruz, sc-52701),IL-1β/IL-1F2(RnDSystems,AF-401-SP), F4/80(Invitrogen,14–4801-82),MFF(Proteintech,17090-1-AP) and MID51(Proteintech, 20164-1-AP).

### Cellular respiration assessment using the Seahorse XF24 Analyzer

Mitochondrial respiration was assessed using a Seahorse XF24 extracellular flux analyzer (Seahorse Bioscience, Copenhagen, Denmark). Cells were seeded in Seahorse XF24 cell culture microplates and treated 24 h before analysis. The analysis was conducted in growth media supplemented with 1 mM sodium pyruvate, 1 mM glutamine, and 10 mM glucose. After a 1-h acclimatization period, basal oxygen consumption was recorded for 20 min. Subsequently, OCR measurements were taken following the sequential addition of mitochondrial inhibitors: (1) oligomycin (1.5 mM) to evaluate ATP synthesis coupling efficiency; (2) the uncoupler carbonyl cyanide 4-trifluoromethoxy-phenylhydrazone (0.5 mM) to assess spare respiratory capacity; and (3) a combination of rotenone (0.5 mM) and antimycin A (0.5 mM) to measure nonmitochondrial consumption of O_2_.

### Statistical analyses

ANOVA was utilized for multigroup comparison, and the t test was employed to demonstrate a difference between two groups that was statistically significant. For one-way ANOV A and two-way ANOVA, respectively, the Fisher least significant difference test and the Dunn multiple comparisons test were applied. P values less than 0.05 were deemed significant. The data was presented as mean ± SD. All computations were performed using GraphPad Prism 9.

## Results

### The activation of Drp1 and mitochondrial fragmentation are early events in renal IRI

**Fig. 1:**
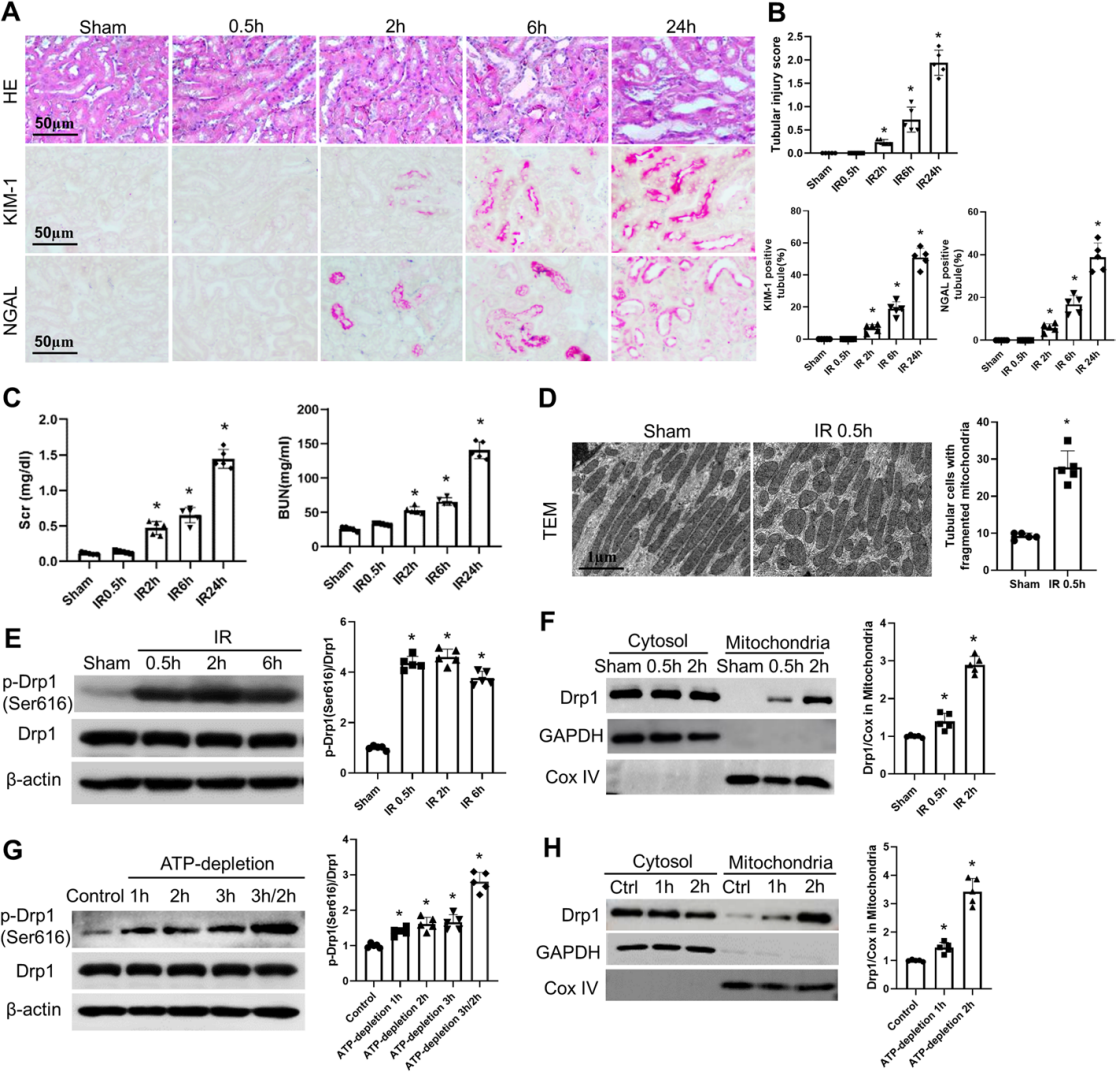
The activation of Drp1 and mitochondrial fragmentation are early events in renal IRI. **A** Representative images of H&E staining, KIM-1 and NGAL immunohistochemistry, Scale bar = 50 µm. **B** Pathological score of tubular damage, quantification of KIM-1 and NGAL positive tubules. **C** The serum creatinine level and BUN level. **D** Representative electron micrographs of mitochondrial morphology in proximal tubule cells. **E** Representative Immunoblot and quantitative analysis of p-Drp1(Ser616) in kidney tissue. β-actin was used as a loading control. **F** Representative Immunoblot and densitometry analysis of Drp1 in renal cytosolic and mitochondrial fractions. COX IV and glyceraldehyde-3-phosphate dehydrogenase (GAPDH) were used as loading controls of mitochondrial and cytosolic fractions, respectively. **G** Representative Immunoblot and quantitative analysis of P-Drp1(Ser616) in mPTCs cells.β-actin was used as a loading control. **H** Representative Immunoblot and densitometry analysis of Drp1 in mPTCs cells cytosolic and mitochondrial fractions. COX IV and glyceraldehyde-3-phosphate dehydrogenase (GAPDH) were used as loading controls of mitochondrial and cytosolic fractions, respectively. Quantitative data are expressed as mean ± SD (*n* = 5). *P < 0.05 versus respective Sham group or Control group

We subjected mice to bilateral renal ischemia by clamping the renal arteries for 28 min, followed by reperfusion for different durations. After 2 and 6 h of reperfusion, there was a mild increase in serum creatinine and blood urea nitrogen levels, as well as the onset of pathological damage to renal tubules (Fig. 1A, B). Furthermore, after 24 h of reperfusion, the levels of creatinine and blood urea nitrogen, as well as the severity of renal pathological damage, became more pronounced (Fig. 1C). Interestingly, fragmentation of mitochondria (Fig. 1D), as well as phosphorylation of Drp1 at the serine 616 residue and translocation of Drp1 to mitochondria (Fig. 1E, F), were observed as early as 30 min after reperfusion initiation. However, at this time point, there was no apparent pathological damage or renal functional abnormalities (Fig. 1A). Subsequently, in our in vitro model, we induced ATP depletion in renal tubular epithelial cells to simulate IRI. Cells subjected to ATP depletion for 3 h, followed by 2 h of recovery, exhibited significant cell death. On the other hand, ATP depletion for 1 h did not cause noticeable cell death but resulted in the translocation of Drp1 to mitochondria and alterations in mitochondrial morphology (Fig. 1G, H). These combined in vivo and in vitro experimental results suggest that changes in mitochondrial morphology and activation of Drp1 are early events in renal ischemia–reperfusion injury.

### P110 alleviates ischemic acute injury and Inflammatory response in mice

**Fig. 2:**
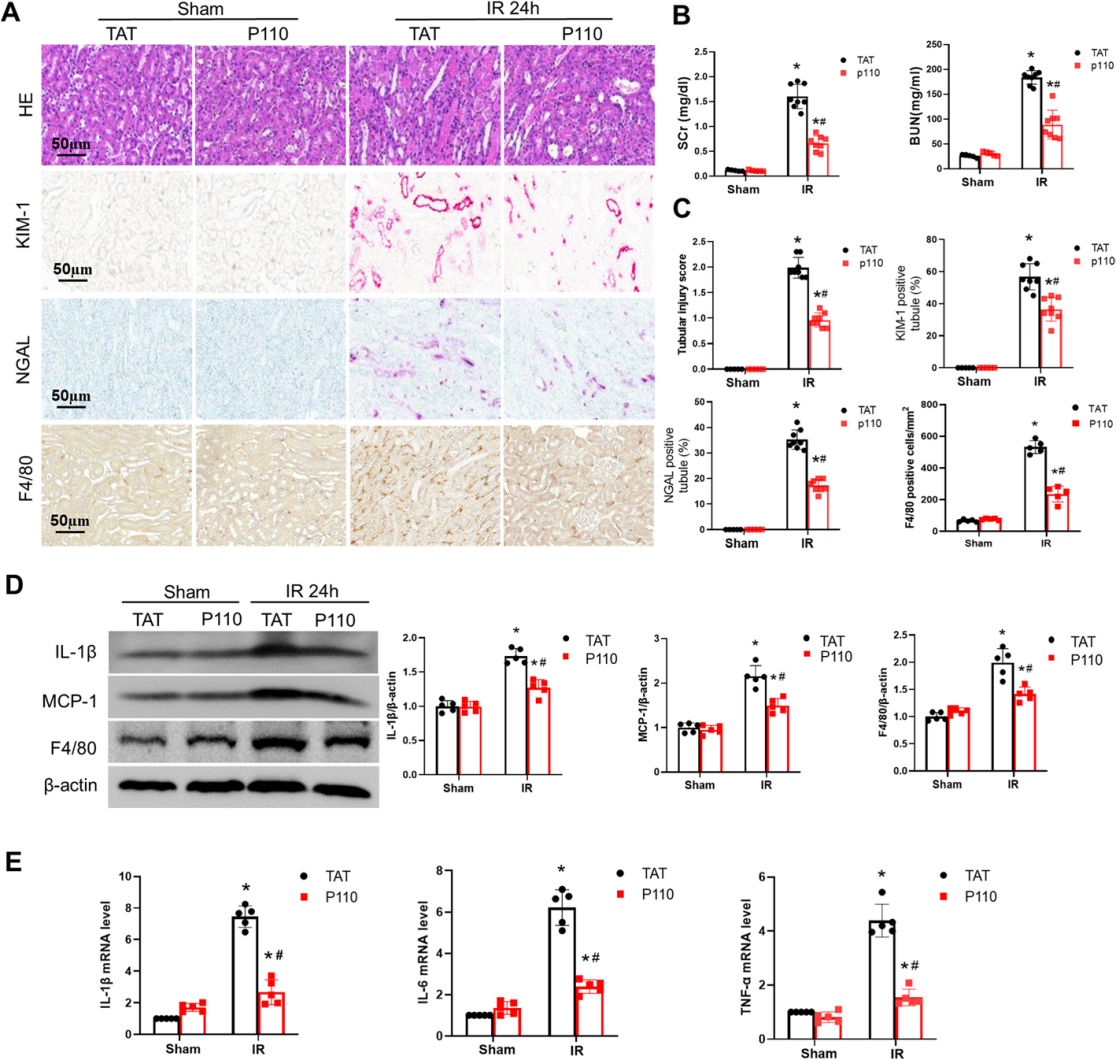
P110 alleviates ischemic AKI and Inflammatory response in mice. **A** Representative images of H&E staining, KIM-1,NGAL and F4/80 immunohistochemistry. Scale bar = 50 μm. **B** Serum creatinine and BUN measurement. **C** Pathological score of tubular damage, Quantification of KIM-1,NGAL and F4/80 positive tubules. **D** Representative Immunoblot and quantitative analysis of IL-1β,MCP-1 and F4/80 in renal.β-actin was used as loading control. **E** q-PCR analysis of IL-1β, IL-6 and TNF-α. Quantitative data are expressed as mean ± SD (*n* = 5–8). *P < 0.05 versus respective Sham group; ^#^*P* < 0.05 versus IR group

Mdivi1, used as a Drp1 inhibitor, has been demonstrated to exert a protective effect against renal IRI [14, 30]. However, its specificity has recently been challenged [25]. On the other hand, P110, a specific Drp1 inhibitor [15, 32], has been shown to have a protective effect in neurodegenerative diseases and cardiac dysfunction [15, 33]. We investigated the role of P110 in renal IRI. Based on previous literature, we chose to administer the drug twice at 1 day and 30 min before surgery, using intraperitoneal injections at doses of 0.1 mg/kg, 0.5 mg/kg, 1 mg/kg, and 2 mg/kg. Renal function analysis revealed that injection of P110 resulted in a concentration-dependent reduction in post-IRI creatinine and blood urea nitrogen levels (0.1 mg/kg to 1 mg/kg), with no further increase in protection observed at 2 mg/kg (Additional file 1: Fig S1B). At a concentration of 1 mg/kg, we examined different timing regimens, including daily administration for 4 days before surgery, administration 1 day before surgery, and administration 12 h after surgery (Additional file 1: Fig S1B). No significant differences in renal function were observed between administration 1 day before and administration for 4 days before surgery. The therapeutic effect was diminished when administered only 12 h after surgery (Additional file 1: Fig S1B). Therefore, we primarily employed a pre-treatment strategy of administering 1 mg/kg of P110 1 day and 30 min before surgery in subsequent experiments. Histology and renal function analysis demonstrated that P110 (1 mg/kg) reduced renal pathological damage (Fig. 2A, B) and renal function impairment (Fig. 2C). Immunohistochemical staining of injury markers, KIM-1 and NGAL, in the proximal and distal tubules of the kidney revealed that P110 was capable of concurrently reducing the expression of KIM-1 and NGAL induced by IRI (Fig. 2A). P110 also demonstrated the ability to reduce macrophage infiltration induced by IRI. (Fig. 2A, B). Additionally, qPCR results and immunoblot results demonstrated that P110 reduced the expression of inflammatory factors IL-6, IL-1β, and TNF-α following IRI (Fig. 2D, E).

### P110 outperforms Mdivi-1 in renal IRI treatment with no apparent toxic effects at high doses

We further compared the effects of Mdivi-1 and P110 on renal IRI. The commonly used dose for Mdivi-1 is 50 mg/kg [14, 34], and we selected three doses: 25 mg/kg, 50 mg/kg, and 75 mg/kg, intraperitoneally. Interestingly, Mdivi1 (50 mg/kg and 75 mg/kg) was also able to alleviate renal IRI, however its therapeutic effect was not as pronounced as that of P110 (Additional file 4: Fig S4A–D). Furthermore, we conducted a drug toxicity experiment using a high dose of 100 mg/kg P110, administered continuously for 4 days in mice. The mice exhibited no substantial alterations in body weight, liver and kidney function, electrolyte levels, or activity levels (Additional file 2: Fig S2A–D). Furthermore, the histology of heart, liver, lung, spleen, kidney, and bone tissues remained unaltered following P110 treatment, as evidenced by their normal morphology (Additional file 3: Fig S3), thereby underscoring the safety profile of P110. Taken together, these findings suggest that P110 is a safe and effective agent for mitigating renal IRI by attenuating the associated damage.

### P110 enhances mitochondrial recovery and cell proliferation

**Fig. 3:**
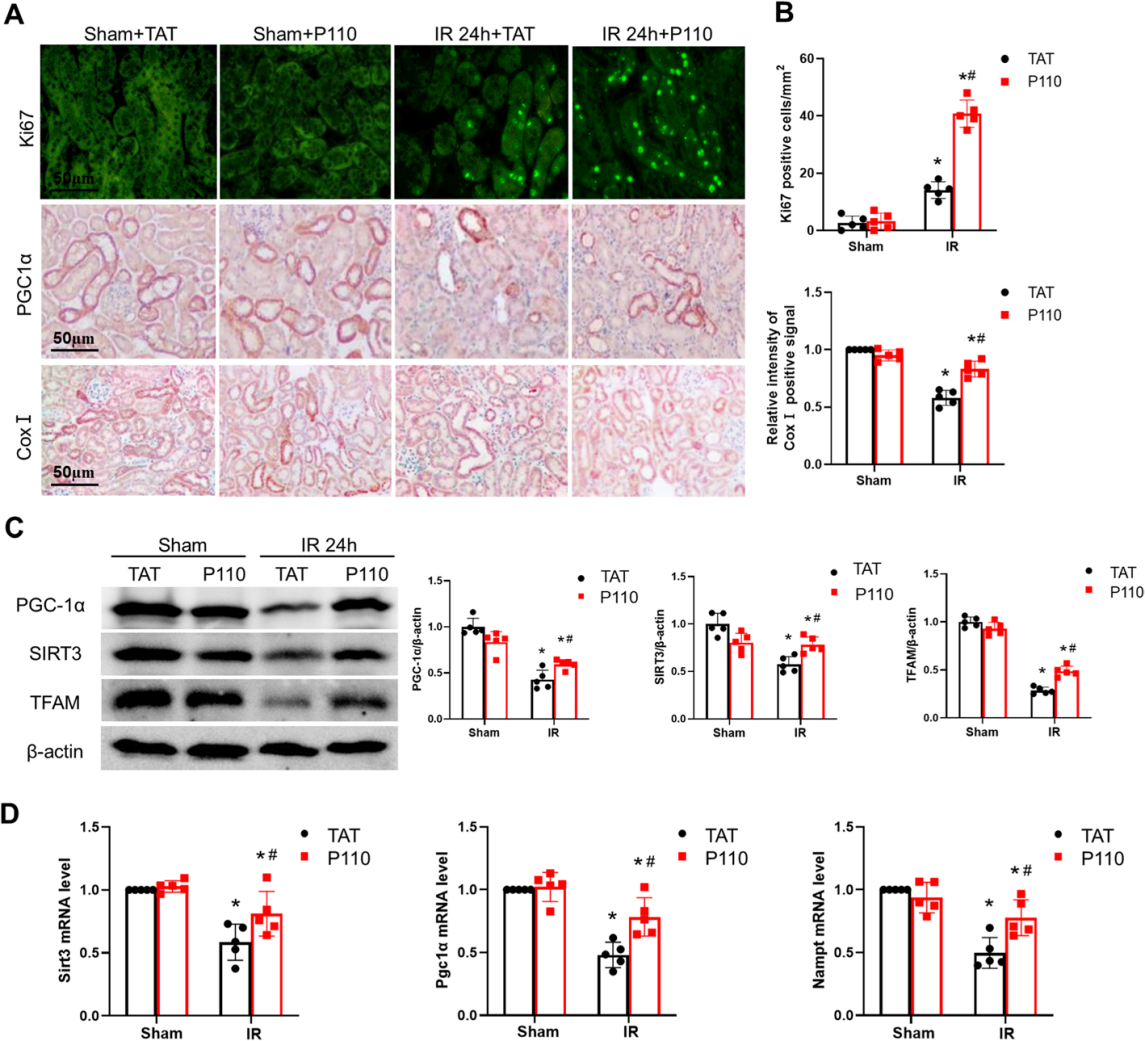
P110 enhances mitochondrial recovery and cell proliferation. **A** Representative images of Ki67 immunofluorescence and PGC-1α and Cox I immunohistochemistry. Scale bar = 50 μm. **B** Quantification of Ki67 and Cox I positive tubules. **C** Representative Immunoblot and quantitative analysis of PGC-1α,SIRT3 and TFAM.β-actin was used as a loading control. **D** q-PCR analysis of Sirt3,Pgc1α and Nampt. Quantitative data are expressed as mean ± SD (*n* = 5). *P < 0.05 versus respective Sham group; ^#^*P* < 0.05 versus IR group

The restoration of mitochondrial function after ischemia is of utmost importance for kidney repair[35]. Ki67 upregulation serves as one of the markers for activated tubular cell proliferation after ischemia, and immunofluorescence staining demonstrated increased expression of the cell proliferation marker Ki67 following P110 treatment (Fig. 3A, B). Immunohistochemistry for mitochondrial cytochrome c oxidase I revealed that mitochondrial content was preserved after IRI and subsequent P110 treatment (Fig. 3A, B). Furthermore, immunohistochemistry staining revealed the restoration of PGC-1α, a primary regulator of mitochondrial biogenesis and function, after P110 treatment (Fig. 3A). Additionally, we conducted assessments of genes and proteins related to mitochondrial function, and observed that the protein levels of PGC-1α, SIRT3, and TFAM and the mRNA levels of Sirt3, Pgc-1α, and Nampt were restored in kidney tissues following intervention with P110 (Fig. 3C, D). The expression of these genes is critically important for kidney repair.

### P110 reduced mitochondrial dysfunction in the ATP depletion model and enhanced cellular vitality

**Fig. 4:**
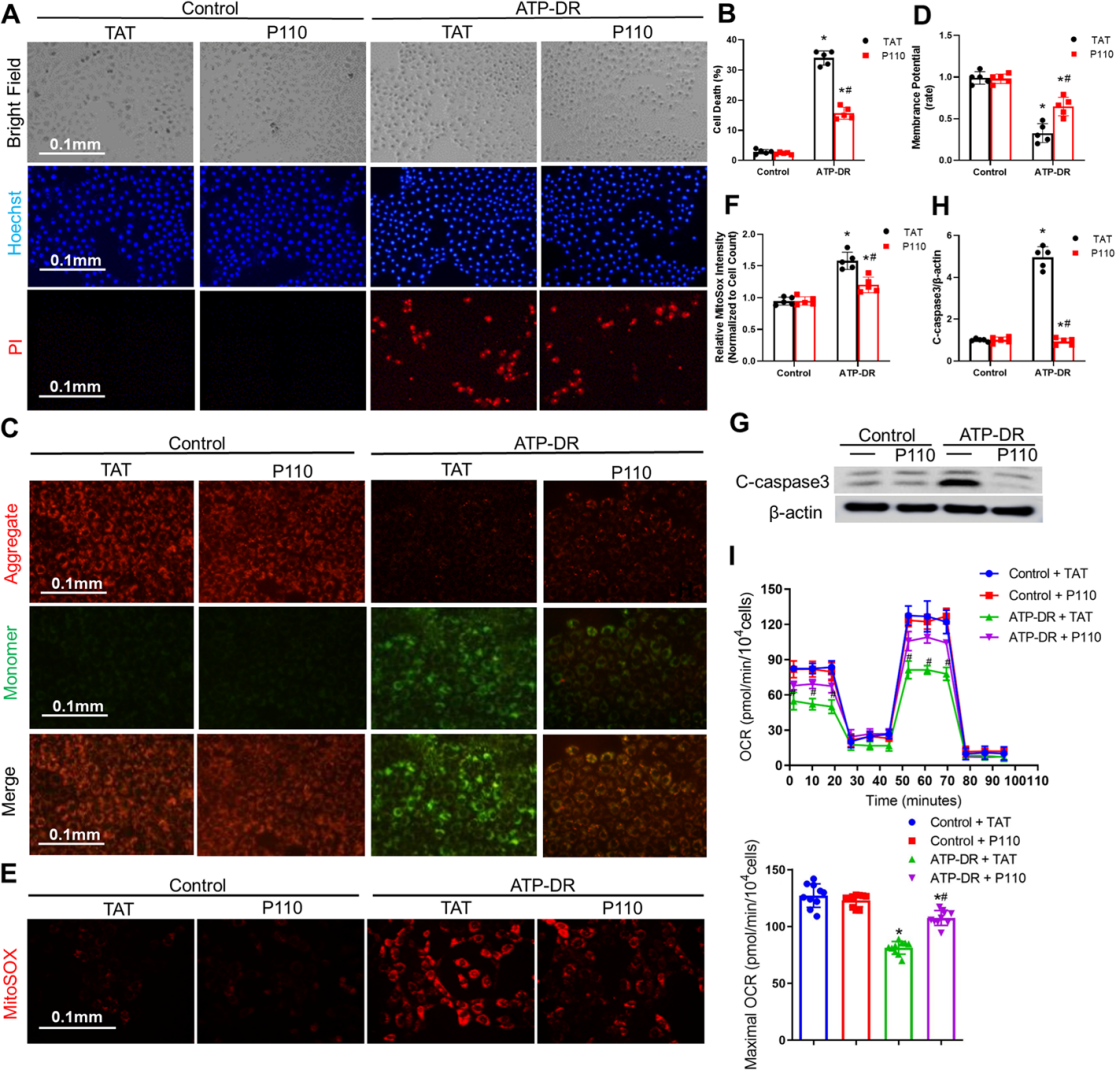
P110 reduced mitochondrial dysfunction in the ATP depletion model and enhanced cellular vitality **A** Representative images of cell morphology, DAPI staining of cell nuclei, PI staining and Merge. Scale bar = 100 μm. **B** Percentage of cell death assessed morphologically. **C**, **D** Representative images and quantification of JC-1 staining showing red fluorescence of JC-1 aggregate and green signal of monomer. **E**, **F** mPTCs were incubated with 5 μM CellROX Deep Red reagent for 30 min. ROS generation was visualized by fluorescence microscopy. **G**, **H** Representative Immunoblot and quantitative analysis of Cleaved-caspase3.β-actin was used as a loading control. **I** Measurement of the mitochondrial oxygen consumption ratio (OCR) of mPTCs cells treated with ATP depletion for 3 h compared to control cells (*n* = 10 cultures per treatment group). Maximal OCR were mentioned. Quantitative data are expressed as mean ± SD. *P < 0.05 versus respective Control group; ^#^*P* < 0.05 versus ATP-DR group

We employed ATP depletion recovery model (ATP-DR) to induce renal tubular cell injury, mimicking the in vivo IRI condition[10, 31]**.** PI and Hoechst staining demonstrated that P110 alleviated cell death induced by ATP-DR (Fig. 4A, B). Furthermore, immunoblot analysis of cleaved caspase-3 indicated that P110 can reduce cell apoptosis caused by ATP-DR (Fig. 4G, H). The production of mitochondrial superoxide (O2 –), a primary source of mitochondrial ROS (mitoROS), significantly increased after ATP-DR, and this increase was effectively mitigated by P110 treatment (Fig. 4E, F). JC-1 staining showed that ATP-DR led to a transition from red fluorescence to green fluorescence, indicating a decrease in membrane potential, while P110 improved the reduction in membrane potential induced by ATP-DR (Fig. 4C, D). Oxygen consumption rate revealed that under normal conditions, P110 did not impact cellular respiration, but it significantly enhanced mitochondrial respiratory function after ATP-DR (Fig. 4I). Consequently, it is evident that P110 can alleviate renal tubular cell injury induced by ATP depletion.

### P110 reduces the translocation of Drp1 to the mitochondria and mitochondrial fission

**Fig. 5:**
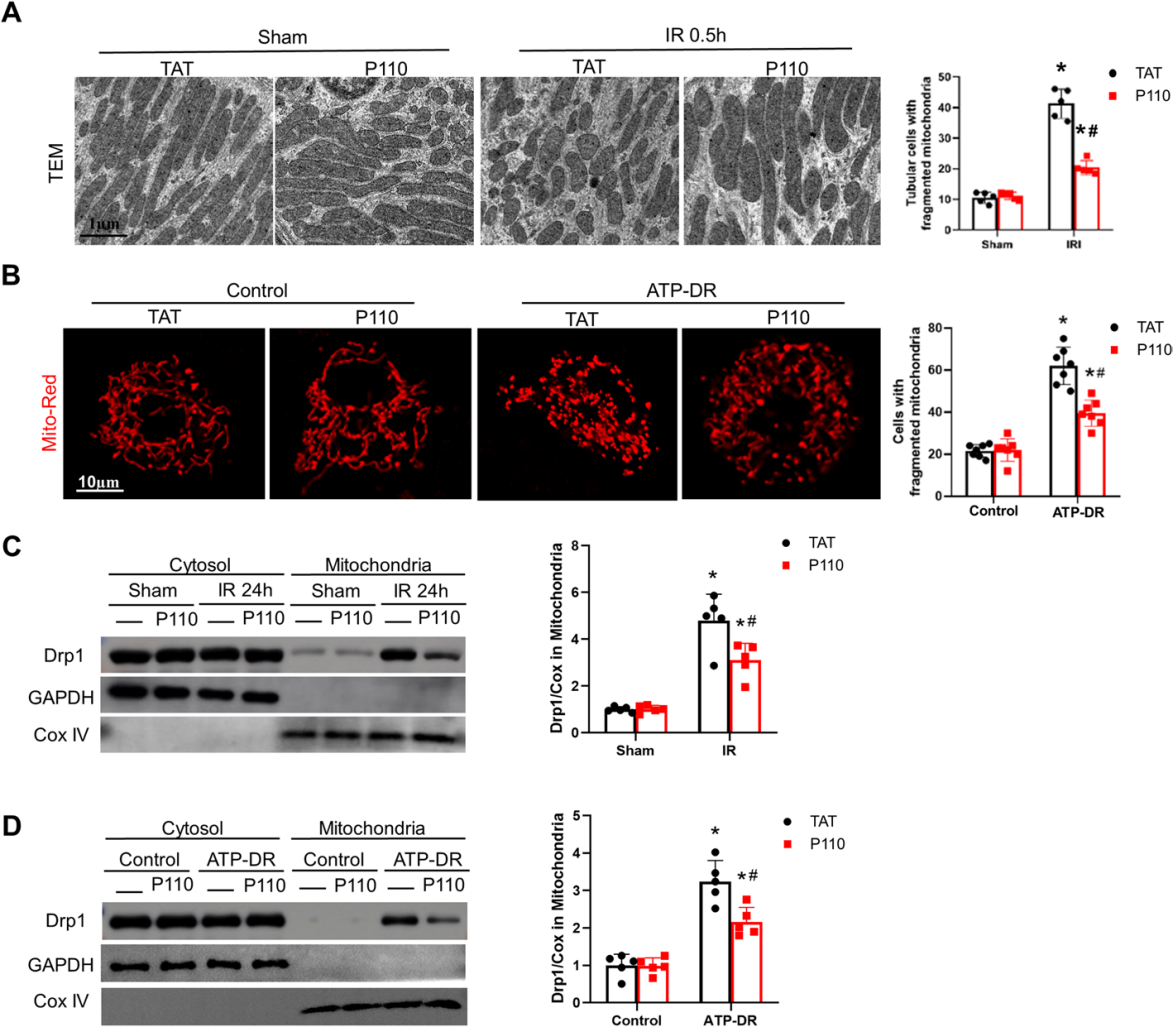
P110 reduces the translocation of Drp1 to the mitochondria and mitochondrial fission. **A** Representative electronic microscopy images of proximal tubular cells and percentage of proximal tubule cells with mostly fragmented mitochondria (1% filamentous mitochondria). **B** Fluorescence after mitoRed transfection and the number of cells with fragmented mitochondria was quantified in each condition. **C** Representative Immunoblot and densitometry analysis of Drp1 in renal cytosolic and mitochondrial fractions. COX IV and glyceraldehyde-3-phosphate dehydrogenase (GAPDH) were used as loading controls of mitochondrial and cytosolic fractions, respectively. **D** Representative immunoblots densitometry analysis of Drp1 in mPTCs cells cytosolic and mitochondrial fractions. COX IV and glyceraldehyde-3-phosphate dehydrogenase (GAPDH) were used as loading controls of mitochondrial and cytosolic fractions, respectively. Quantitative data are expressed as mean ± SD. *P < 0.05 versus respective Sham/Control group; ^#^*P* < 0.05 versus IR/ATP-DR group

Electron microscopy (EM) results revealed that mitochondria in mouse kidneys displayed fragmentation as early as 30 min after reperfusion. Interestingly, the use of P110 significantly mitigated the mitochondrial fragmentation induced by IRI (Fig. 5A). Fluorescence confocal microscopy similarly showed that mitochondria transformed from a filamentous to a fragmented state after ATP-depletion treatment, and the addition of P110 led to a partial restoration of mitochondrial morphology (Fig. 5B). The translocation of Drp1 to mitochondria and excessive mitochondrial fission are crucial factors in kidney IRI. Our research findings indicate that treatment with P110 significantly reduces the translocation of Drp1 to mitochondria in both tissue and cells (Fig. 5C–D), thereby highlighting P110's capability to diminish mitochondrial division induced by AKI.

### P110 reduces the interaction between Fis1 and Drp1 induced by ATP depletion

**Fig. 6:**
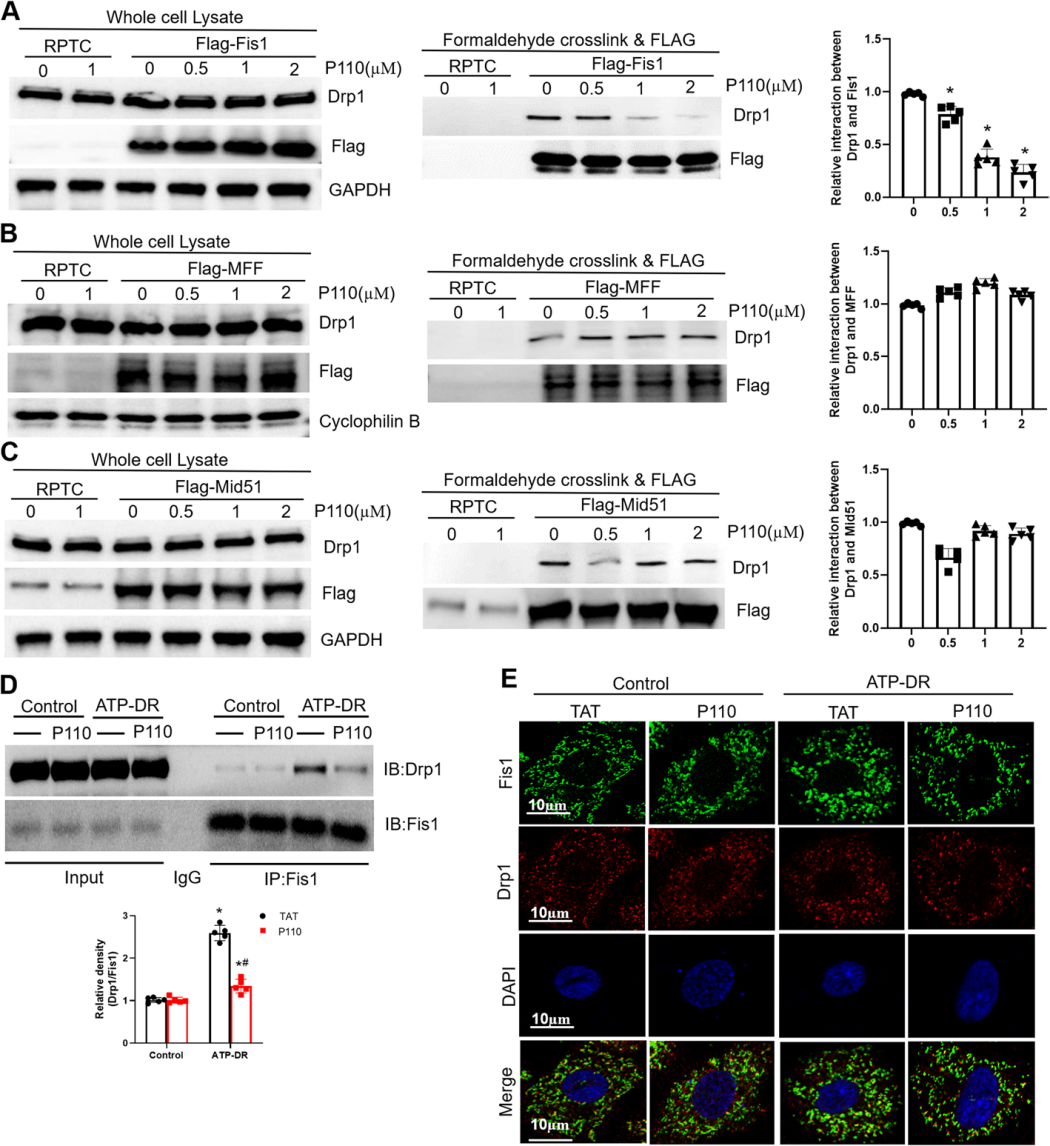
P110 reduces the interaction between Fis1 and Drp1 induced by ATP depletion. **A**–**C** Immunoprecipitation analysis of the Fis1-Drp1 interaction 、MFF-Drp1 interaction and the MiD51-Drp1 interaction. **E** Representative confocal fluorescence microscopy of Drp1 and Fis1 in mPTC cells. Scale bar = 10 μm. **D** mPTC cells were treated with the P110 (1 µM) for 3 h followed by treatment with ATP-depletion for 2 h. Following a brief cross-linking, cells were homogenized. Total cell lysates were then subjected to immunoprecipitation (IP) with anti-Fis1 antibodies, and the immunoprecipitates were analyzed by immunoblotting (IB) with anti-Drp1antibodies. Quantification of Drp1/Fis1 expression. Quantitative data are expressed as mean ± SD. *P < 0.05 versus respective Control group;#, P < 0.05 versus ATP-DR group

The translocation of Drp1 to mitochondria involves its binding to mitochondrial receptors MFF, Mid51 (Mid49), and Fis1, which are proteins located on the outer mitochondrial membrane responsible for mitochondrial fission[36]. To elucidate the specific mechanisms of P110 in renal tubular injury, we examined the interactions of Drp1 with Fis1, Mid51, and MFF in the presence of P110. Immunoprecipitation experiments indicated that ATP depletion promoted the interaction between Fis1 and Drp1, while P110 treatment reduced the interaction between Fis1 and Drp1 (Fig. 6A), without affecting the binding of Drp1 with MFF and Mid51 (Fig. 6B, C). Furthermore, we similarly observed that P110 decreased the binding between Drp1 and Fis1 using endogenous Drp1 and Fis1 antibodies (Fig. 6D). Additionally, using laser confocal fluorescence microscopy, we observed that under control conditions, there was relatively minimal colocalization of green fluorescence from Fis1 and red fluorescence from Drp1. However, under ATP-depletion conditions, the colocalization of Fis1 and Drp1 increased, and this interaction was significantly reduced after treatment with P110 (Fig. 6E).

### P110 reduces the translocation of Bax induced by ATP depletion and the release of mtDNA

**Fig. 7:**
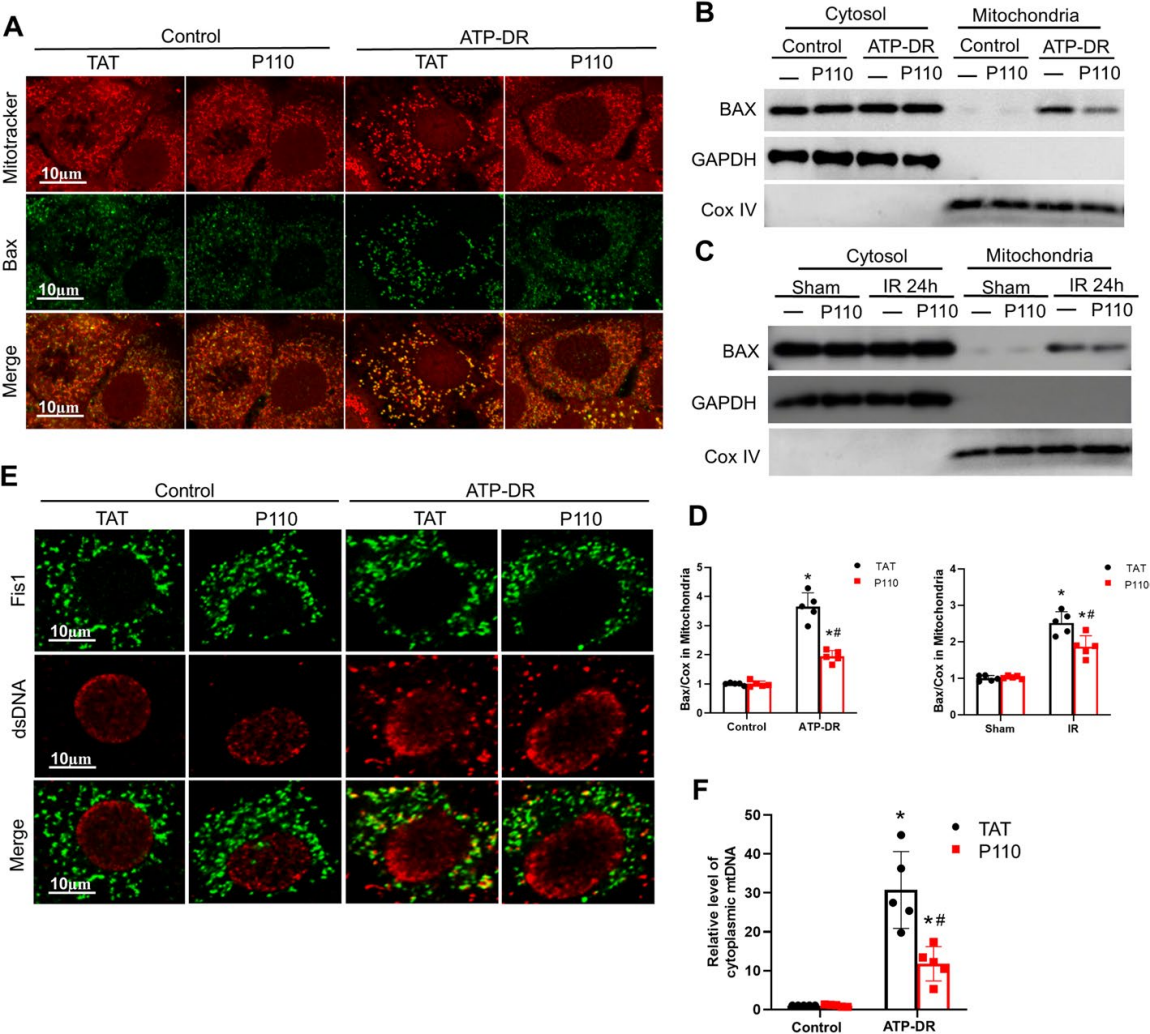
P110 reduces the translocation of Bax induced by ATP depletion and the release of mtDNA. **A** Representative confocal fluorescence microscopy of Bax and Mitotracker in mPTC cells. Scale bar = 10 μm **B**–**D** Representative Immunoblot and densitometry analysis of Bax in renal cytosolic and mitochondrial fractions. COX IV and GAPDH were used as loading controls of mitochondrial and cytosolic fractions, respectively. **E** Representative confocal fluorescence microscopy of dsDNA and Fis1 in mPTC cells. Scale bar = 10 μm. **F** Ratio of cytoplasmic to total mtDNA determined by qPCR analysis of mPTC cells. Quantitative data are expressed as mean ± SD. *P < 0.05 versus respective Sham/Control group; ^#^*P* < 0.05 versus IR/ATP-DR group

Mitochondrial fragmentation exacerbates the recruitment of Bax to mitochondria[37], leading to alterations in outer membrane permeability and subsequent leakage of mitochondrial DNA (mtDNA), triggering the innate immune cGAS/STING pathway[38]. Immunohistochemical validation in human AKI samples confirmed the upregulation of cGAS and STING[39].In both in vivo and in vitro experiments, we observed the accumulation of Bax in mitochondria following injury, while the P110 treatment resulted in reduced translocation of Bax to mitochondria (Fig. 7A–D). The release of mitochondrial DNA is a critical event in the activation of the cGAS/STING pathway upon mitochondrial damage. Using laser confocal fluorescence microscopy, we observed increased co-localization of dsDNA and Fis1 following ATP-DR, which was attenuated by the addition of P110 (Fig. 7E, F).

### P110 alleviates the cGAS-STING pathway activated by mtDNA

**Fig. 8:**
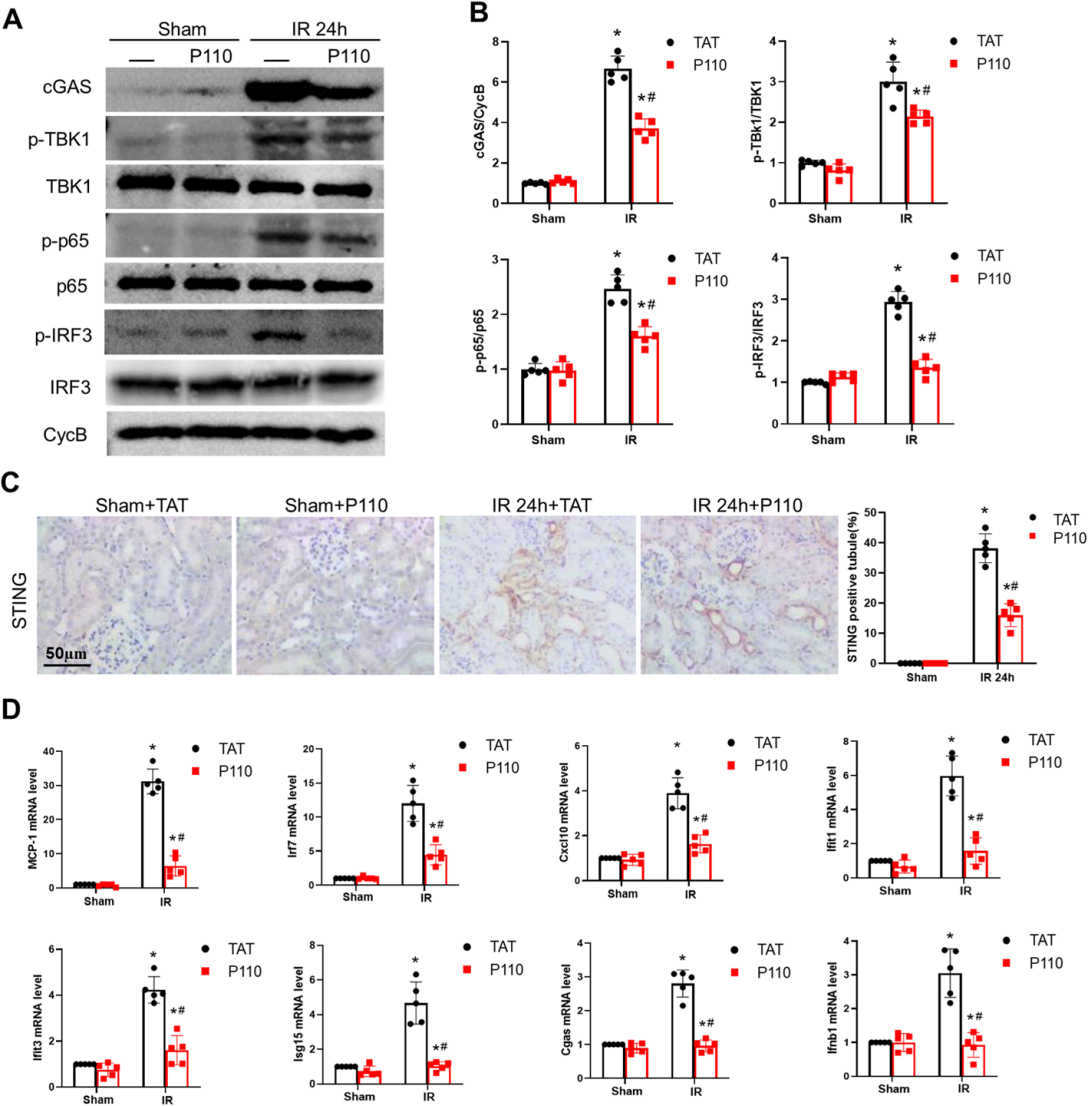
P110 alleviates the cGAS-STING pathway induced by IRI. **A**, **B** Representative Immunoblot and quantitative analysis of cGAS,p-TBK1,p-p65 and p-IRF3 in kidney tissue. Quantitative data represent the relative ratio to total TBK1 or p65 or IRF3.Cyclophilin B (CycB) was used as loading control. **C** Representative images of STING immunohistochemistry. Scale bar = 50 μm. **D** q-PCR analysis of MCP-1, Irf7,Cxcl10,Ifit1,Ifit3,Isg15,Cgas and Ifnb1.Quantitative data are expressed as mean ± SD. *P < 0.05 versus respective Sham group; ^#^*P* < 0.05 versus IR group

Dysfunctional mitochondria can activate the cGAS-STING pathway by releasing mtDNA, leading to increased phosphorylation of TBK1 and p65 and subsequent initiation of sterile inflammation. We observed that ischemic AKI triggers the release of mtDNA. Both tissue and cellular responses to injury stimuli exhibited increased expression of cGAS-STING, followed by activation of p-TBK1、p-p65 and p-IRF3. However, the addition of P110 inhibits the cGAS-STING-p-TBK1、p-p65 and p-IRF3 pathway **(**Fig. 8A–C). Furthermore, IRI upregulated inflammatory factors associated with the cGAS-STING pathway, including MCP-1, Irf7, Cxcl10, Ifi44, Ifit1, Ifit3, Isg15, and Cgas, as evidenced by increased mRNA expression. However, treatment with P110 reduces the elevation of these inflammatory factors (Fig. 8D). Furthermore, P110 treatment alleviated cGAS-STING pathway under ATP-DR condition (Additional file 6: Fig S6). These results suggest that P110 can alleviate sterile inflammation mediated by the cGAS-STING pathway.

### P110 alleviates renal IRI in Bama pigs and folic acid-induced nephropathy in mice

**Fig. 9:**
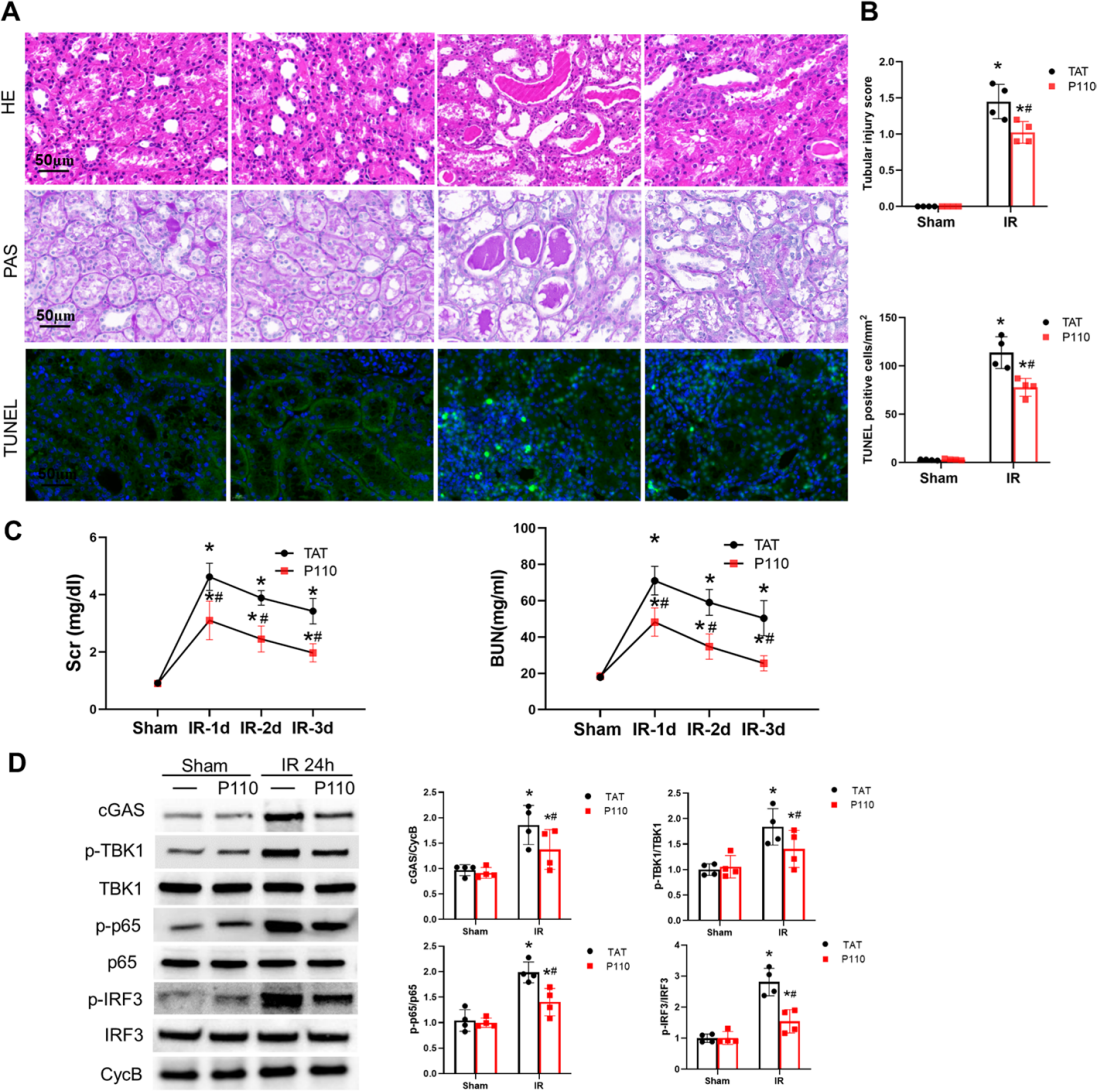
P110 alleviates ischemic AKI and inflammation in Bama pigs. **A** Representative images of H&E staining, PAS staining and TUNEL staining, Scale bar = 50 µm. **B** Pathological score of tubular damage and Quantitative analysis of apoptotic cell numbers per mm square of kidney tissue. **C** The serum creatinine level. **D** The BUN level. **D** Representative Immunoblot and quantitative analysis of cGAS,p-TBK1,p-p65 and p-IRF3 in Bama pigs kidney tissue. Quantitative data represent the relative ratio to total TBK1 or p65 or IRF3. Cyclophilin B (CycB) was used as loading control.Quantitative data are expressed as mean ± SD. *P < 0.05 versus respective Sham group; ^#^*P* < 0.05 versus IR group

To validate the effectiveness of P110 and to mimic human physiological conditions, we utilized Bama pigs to induce unilateral renal ischemia–reperfusion injury. Histological and renal function analysis showed that P110 treatment (0.4 mg/kg) alleviated renal pathological damage (Fig. 9A , B) and renal function damage (Fig. 9C). In addition, changes in the cGAS-STING pathway were also detected, which were consistent with ischemia reperfusion in mice. In Bama pigs, P110 also inhibited the cGAS, p-TBK1, p-p65, and p-IRF3 pathways (Fig. 9D).

We further explored other AKI models, such as folic acid-induced nephropathy. Folic acid was found to induce mitochondrial fragmentation and Drp1 translocation to the mitochondria in renal tubules. Treatment with P110 was capable of reducing mitochondrial fragmentation and Drp1 translocation (Additional file 5: Fig S5 A-D). It was observed that P110 mitigates folic acid-induced renal functional and structural damage, reducing renal tubular injury markers NGAL and KIM-1 (Additional file 5: Fig S5 A-B). In summary, P110 exhibits a robust and significant protective effect in both mice AKI and the Bama pigs renal IRI model.

## Discussion

Emerging evidence suggests that mitochondrial quality control mechanisms, including mitochondria dynamics, play crucial roles in AKI pathophysiology. In this study, we have made the following discoveries: (1) The activation of Drp1 occurs in the early stages of renal ischemia–reperfusion injury (IRI), and the Drp1-specific inhibitor P110 mitigates the translocation of Drp1 to mitochondria and excessive mitochondrial fission by inhibiting the interaction between Drp1 and Fis1. (2) Administration of P110 provides a protective effect in alleviating AKI, demonstrating a superior efficacy compared to the conventional Drp1 inhibitor Mdivi1. High-dose continuous administration does not exhibit noticeable toxic effects. (3) During renal IRI, there is a translocation of Bax to mitochondria, release of mtDNA, and activation of the cGAS-STING signaling pathway, all of which are suppressed by P110 treatment. (4) Furthermore, P110 also alleviates IRI in the Bama pigs model and folic acid-induced nephropathy. Our findings elucidate the role and mechanism of P110 in renal IRI and provide potential clinical applicability.

Mitochondria serve as the primary site for ATP generation, calcium homeostasis and signaling regulation, as well as mediation of cellular apoptosis. Thus, the integrity of mitochondrial structure and function is of utmost importance for cellular health control [35]. Mitochondrial fusion and fission exist in a dynamic equilibrium. Previous studies have revealed that mitochondrial fragmentation occurs early during renal IRI (15–30 min after reperfusion), and this rapid mitochondrial fragmentation is primarily attributed to excessive Drp1-mediated fission rather than inadequate fusion [11, 30]. The usage of P110 can prevent Drp1-induced mitochondrial fission. Notably, P110 has demonstrated greater benefits than Mdivi-1 in the context of renal IRI, even when administered just 1 day prior to IRI surgery, and it promotes renal tubular recovery. Furthermore, P110 has exhibited the ability to decrease serum creatinine levels, blood urea nitrogen levels, and improve pathological outcomes in porcine renal IRI. Hence, P110 holds promise for clinical application in the context of AKI.

We aim to enhance the metabolic capacity of the kidneys and improve their tolerance to ischemia by providing additional mitochondrial-targeted drugs. P110 enhances mitochondrial dynamics and has demonstrated protective effects in conditions such as stroke, Parkinson's disease, and septic cardiomyopathy [16, 27]**.** P110 can be delivered across the cell membrane and penetrate the blood–brain barrier when combined with the TAT 47–57 carrier peptide [15, 27], similar to other short TAT 47–57-conjugated peptides. In this study, the dose-dependent improvement in mitochondrial metabolism with P110 enhances the expression levels of Sirt3, PGC-1α, and TFAM, promoting proliferation of renal tubular cells (Fig. 3). The implication of these findings is that maintaining mitochondrial function in post-ischemic kidneys is crucial for optimal renal function. The previous studies have demonstrated that P110 treatment selectively inhibits the interaction between Drp1 and Fis1 [27]. Our research supports that the specific action of P110 alleviates the effects of Drp1 and Fis1. Additionally, P110 does not affect the interaction of Drp1 with any other Drp1 mitochondrial adaptors, such as MFF and Mid51**.** Interestingly, blocking the functions of Drp1 and MFF has been reported to inhibit the physiological functions of Drp1, leading to severe side effects [13]. This explains why even after 5 months of treatment in wild-type mice, no adverse reactions were observed [15]. Due to the pivotal role of Drp1 as a key regulator of mitochondrial fission under normal conditions, it is crucial to determine whether the inhibition of Drp1 by P110 will have an impact on cellular bioenergetics in the short or long term. We used a high dose of P110 (100 mg/kg/day) and found no abnormalities in mouse serum albumin (ALB), aspartate aminotransferase (AST), or creatinine (Cr) levels, and no significant changes in mouse behavior or condition.

Drp1 plays a crucial role in regulating mitochondrial dynamics. Although mitochondrial fragmentation has been observed in renal IRI, and inhibiting mitochondrial fission has a protective effect, long-term suppression of mitochondrial fission may not always be beneficial. For instance, mitophagy requires activation of Drp1 [40]. The long-term impact of Drp1 depletion in renal tubules on the kidney remains inconclusive. In the heart, Drp1-induced mitochondrial fission is important for maintaining cardiac and mitochondrial bioenergetic homeostasis, and inhibiting fission can lead to impaired mitochondrial function and cardiac injury[41]**.** In the liver, genetic knockout of Drp1 can lead to the formation of megamitochondria, impair mitochondrial autophagy, and insufficient fission can result in the accumulation of damaged and dysfunctional mitochondria. Deletion of Drp1 in liver cells exacerbates liver mitochondrial damage, especially in alcoholic liver injury [42]. Knockout of Drp1 alters mitochondrial shape in muscle stem cells, leading to impaired muscle regeneration [43]. The data suggests that short-term, highly selective inhibition of Drp1, rather than long-term sustained inhibition of Drp1, may be the optimal strategy for treating ischemic injuries. Importantly, P110 selectively inhibited pathological rather than physiological mitochondrial fission and fragmentation [27]**.** In this study, we observed that P110 does not affect Drp1 translocation and mitochondrial fission in normal cells and normal kidney tissue, but only prevents excessive mitochondrial fission during AKI. Short-term use of P110 reduces ischemic injuries without affecting the physiological division in the later stages of AKI. Taking into account the vital requirement to uphold a dynamic balance in mitochondrial morphology, P110 selectively inhibits pathological fission, rendering it a potentially safer drug.

An important downstream event of mitochondrial dysfunction is the activation of innate immune response, which is associated with the pathogenesis of IRI [28, 44, 45]. Mitochondrial damage and mtDNA leakage are frequently detected in various human diseases, particularly in cases of acute tissue injury [46, 47]. Detection of circulating free mtDNA has even been considered as a potential biomarker for assessing tissue damage [48]**.** Recently, it has been reported that urinary mtDNA levels are associated with Severity of renal dysfunction and delayed graft function after kidney transplantation [49, 50]**.** As a crucial pathway of innate immunity, the cGAS-STING pathway is genetically programmed to respond to dsDNA. Expression of cGAS and STING is increased in AKI patients [39]. Mechanistically, mitochondrial fragmentation in renal tubular cells following injury promotes Bax insertion [37]**,** which in turn enhances mitochondrial membrane permeability and leads to mtDNA release [38]**.** The release of dsDNA after mitochondrial damage activates the cGAS-STING pathway, promoting AKI [28, 51]**.** We observed that treatment with P110 significantly mitigates Bax insertion and alleviates cGAS-STING signaling, suggesting a potential relationship between pathological mitochondrial fission and the protective effect of P110 with the cGAS-STING pathway.

This study has several limitations. Firstly, we did not examine the distribution and metabolic status of P110 in the animal. Secondly, due to ethical and economic considerations, a limited number of large animals were used. Kidneys from pigs were collected at 72 h post-ischemia, primarily owing to widespread tubular cell injury, resulting in substantial mitochondrial damage. Based on our previous experience [11], electron microscopy evaluation of mitochondrial dynamics was not conducted at this time point.

**Fig. 10:**
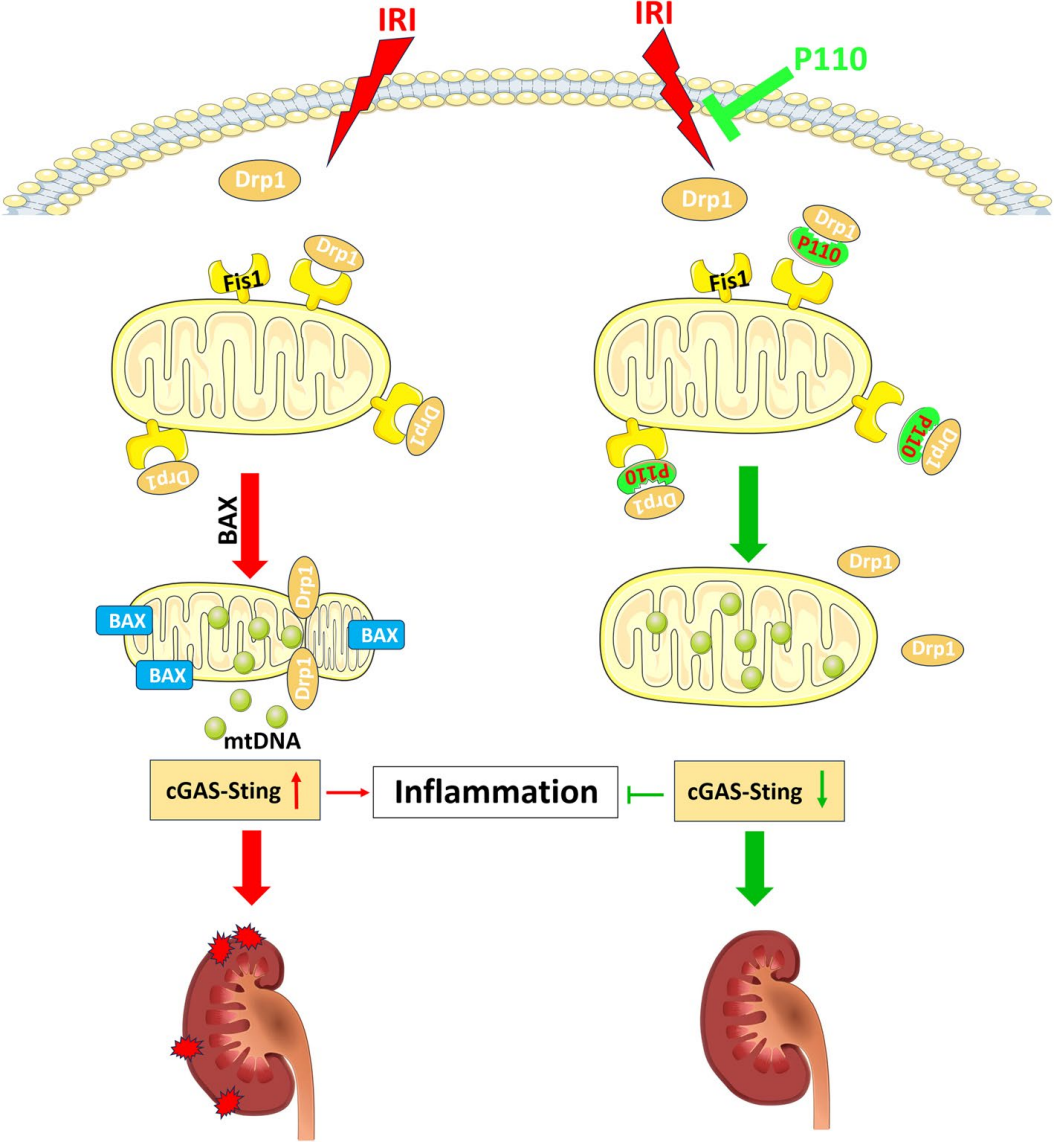
Schematic diagram of the findings of this study. P110 can exert significant protective effects in renal ischemia–reperfusion injury (IRI) by inhibiting the interaction between Drp1 and Fis1, reducing Drp1-mediated mitochondrial fragmentation

In summary, our research results indicate that P110 can exert significant protective effects in murine renal IRI and Bama pig renal IRI by inhibiting the mutual interaction between Drp1 and Fis1, reducing Drp1-mediated mitochondrial fragmentation **(**Fig. 10**)**. Mechanistically, this may occur through the inhibition of Bax-mediated mtDNA release and the aseptic inflammatory cGAS-STING pathway. P110 holds the potential to serve as a clinical intervention targeting mitochondrial dynamics regulation.

## Conclusions

The present findings suggest that P110 can exert significant protective effects in mice renal IRI and Bama pig renal IRI by inhibiting the mutual interaction between Drp1 and Fis1.Nonetheless, the sum of our findings highlights its promise as an innovative treatment for AKI.

### Supplementary Information

**Additional file 1: Figure S1. **Impact of different dosages and administration times of P110 on renal function following renal IRI. **A** The serum creatinine level and BUN level in different drug doses. **B** The serum creatinine level and BUN level in different dosing times. Quantitative data are expressed as mean ± SD. *P < 0.05 versus respective Sham group.

**Additional file 2: Figure S2 **The influence of high-dose P110 on biochemical parameters in mice. **A** The mouse body weight. **B** The ALT level and AST level. **C** The serum creatinine level and BUN level. **D** The serum potassium、sodium and chloride level. Quantitative data are expressed as mean ± SD. *P < 0.05 versus respective Sham + TAT group.

**Additional file 3: Figure S3. **The Impact of High-Dose P110 on the Histopathology of Different Organs. **A** Representative images of H&E staining in heart、lung、kidney、liver、femur and spleen. **B** Representative images of H&E staining in heart、lung、kidney、liver、femur and spleen, Scale bar = 100 µm.

**Additional file 4: Figure S4. **P110 exhibits a better effect against AKI than Mdivi-1. **A** Representative images of H&E staining. Scale bar = 50 µm. Pathological score of tubular damage. **B** Pathological score of tubular damage, Quantification of KIM-1,NGAL and F4/80 positive tubules. **C** The serum creatinine level and BUN level. Quantitative data are expressed as mean ± SD. *P < 0.05 versus respective Sham group. ^#^*P* < 0.05 versus IRI + Vehicle group. ^&^*P* < 0.05 versus IRI + P110 group.

**Additional file 5: Figure S5. **P110 relieves folic acid-induced AKI. **A** The serum creatinine level and BUN level. **B** Representative images of H&E staining,KIM-1 and NGAL immunohistochemistry and electron micrographs of mitochondrial morphology in proximal tubule cells. Scale bar = 50 μm. **C** Pathological score of tubular damage,Quantification of KIM-1 and NGAL positive tubules. **D** Representative Immunoblot and densitometry analysis of Drp1 in renal cytosolic and mitochondrial fractions. COX IV and glyceraldehyde-3-phosphate dehydrogenase (GAPDH) were used as loading controls of mitochondrial and cytosolic fractions, respectively. Quantitative data are expressed as mean ± SD. *P < 0.05 versus respective Sham group; ^#^*P* < 0.05 versus FA group.

**Additional file 6: Figure S6. **P110 alleviates the cGAS-STING pathway induced by ATP-DR. **A**, **B** Representative Immunoblot and quantitative analysis of STING,cGAS,p-TBK1,p-p65 and p-IRF3 in mPTC cells. Quantitative data represent the relative ratio to total TBK1 or p65 or IRF3.Cyclophilin B (CycB) was used as loading control. Quantitative data are expressed as mean ± SD. *P < 0.05 versus respective Control group; ^#^*P* < 0.05 versus ATP-DR group.

**Additional file 7.** Primer sequences used in this study.

## Data Availability

Availability of data and materials All data generated or analyzed during this study are included in this published article.

## Abbreviations

AKI: Acute Kidney Injury
IRI: Ischemia–Reperfusion Injury
ATP-DR: ATP depletion and recovery
Drp1: Dynamin-Related Protein 1
Fis1: Mitochondrial Fission 1
CKD: Chronic kidney disease
ESRD: End-Stage renal disease
MFF: Mitochondrial fission factor
Mid51: Mitochondrial Elongation Factor 1
cGAS: Cyclic GMP-AMP synthase
STING: Stimulator of interferon genes
TUNEL: Terminal deoxynucleotidyl transferase dUTP Nick-End Labeling
HE: Hematoxylin and eosin
ATL: Alaninetransaminase
AST: Aspartate Transaminase
