# Supplementary figures and images for: Inhibition of Drp1- Fis1 interaction alleviates aberrant mitochondrial fragmentation and acute kidney injury
Source: Cell Mol Biol Lett. 2024 Mar 4;29:31. doi: 10.1186/s11658-024-00553-1 (PMC10910703; doi:10.1186/s11658-024-00553-1)

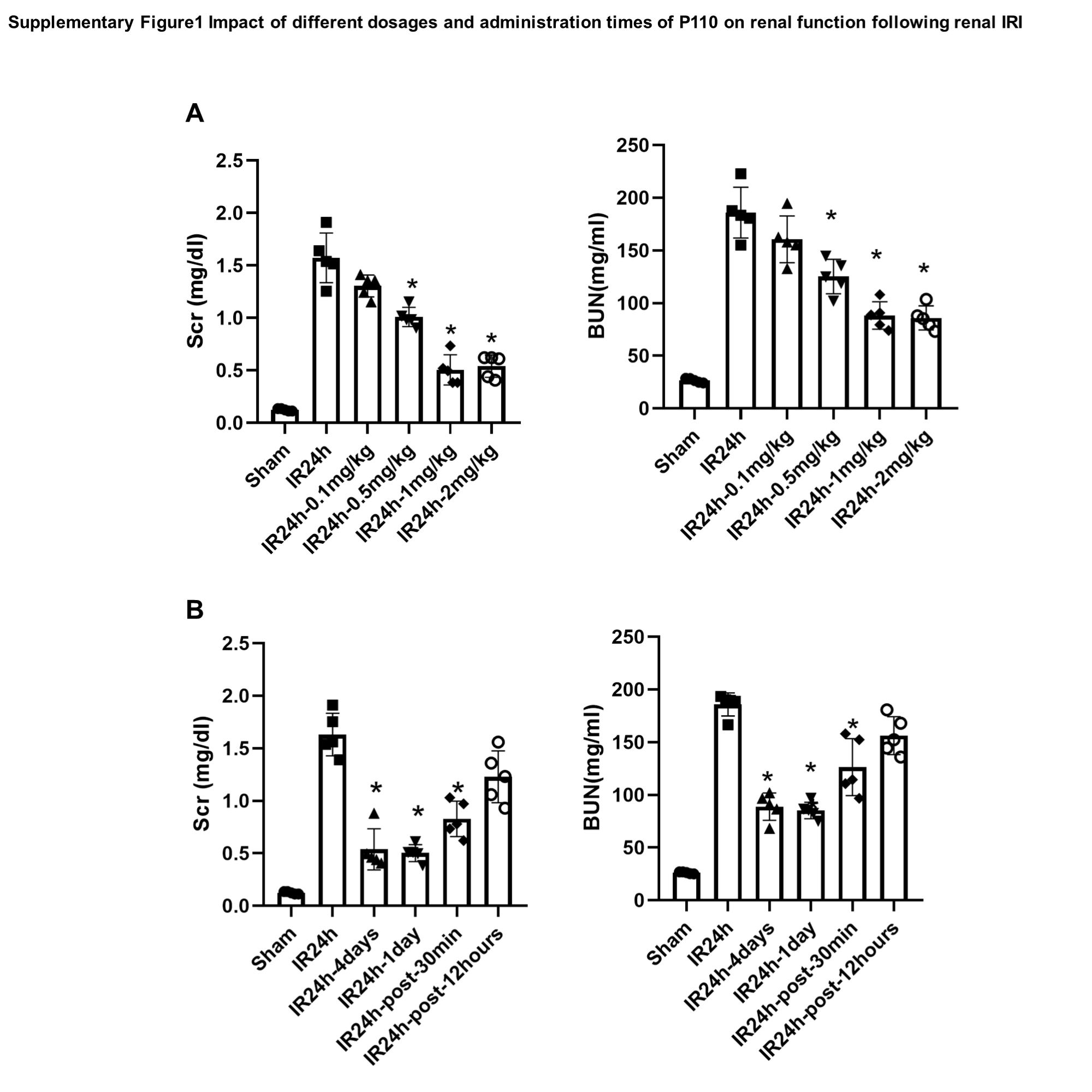

Supplement: Supplementary file 1 — Additional file 1: Figure S1. Impact of different dosages and administration times of P110 on renal function following renal IRI. A The serum creatinine level and BUN level in different drug doses. B The serum creatinine level and BUN level in different dosing times. Quantitative data are expressed as mean ± SD. *P < 0.05 versus respective Sham group. [file 11658_2024_553_MOESM1_ESM.tif]

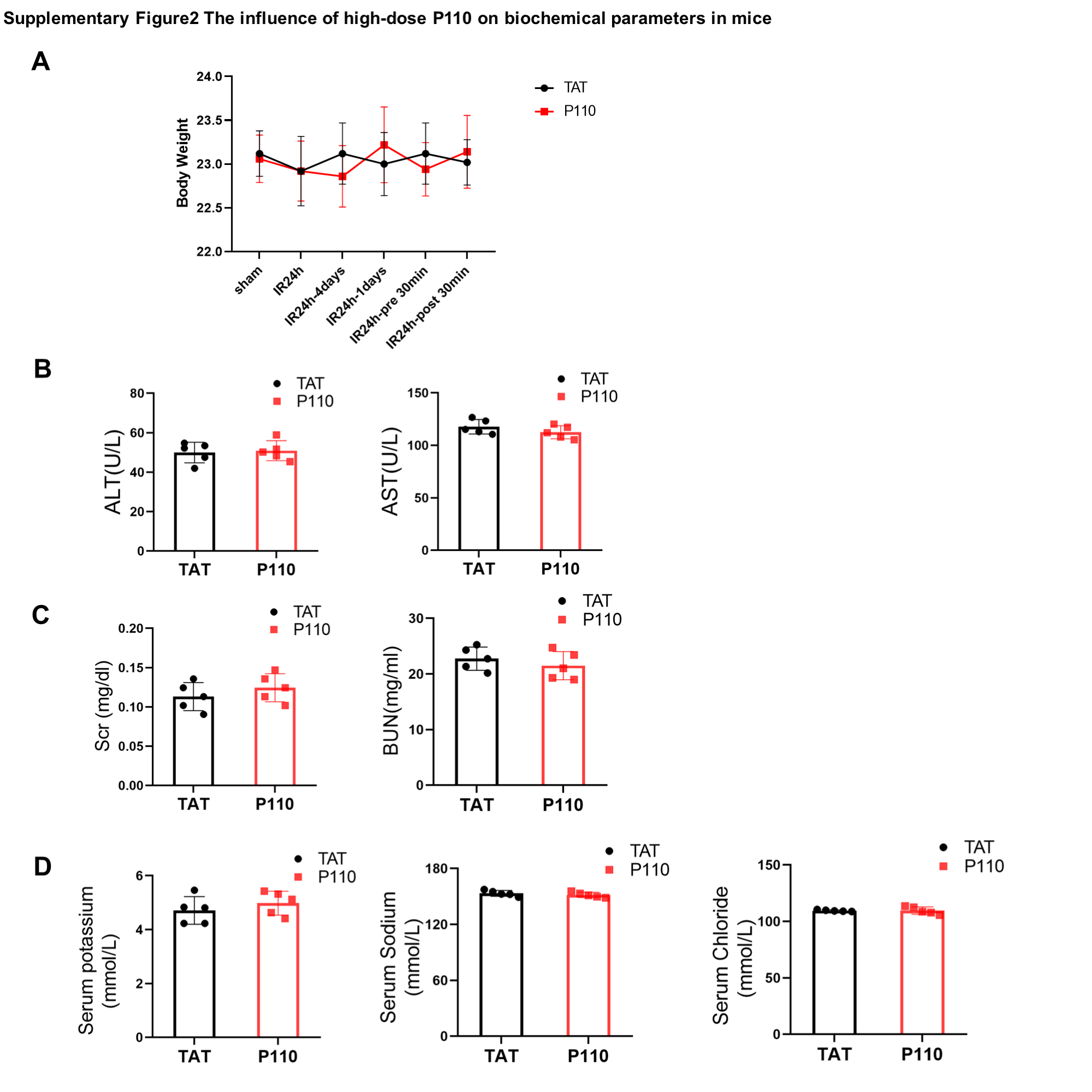

Supplement: Supplementary file 2 — Additional file 2: Figure S2 The influence of high-dose P110 on biochemical parameters in mice. A The mouse body weight. B The ALT level and AST level. C The serum creatinine level and BUN level. D The serum potassium、sodium and chloride level. Quantitative data are expressed as mean ± SD. *P < 0.05 versus respective Sham + TAT group. [file 11658_2024_553_MOESM2_ESM.tif]

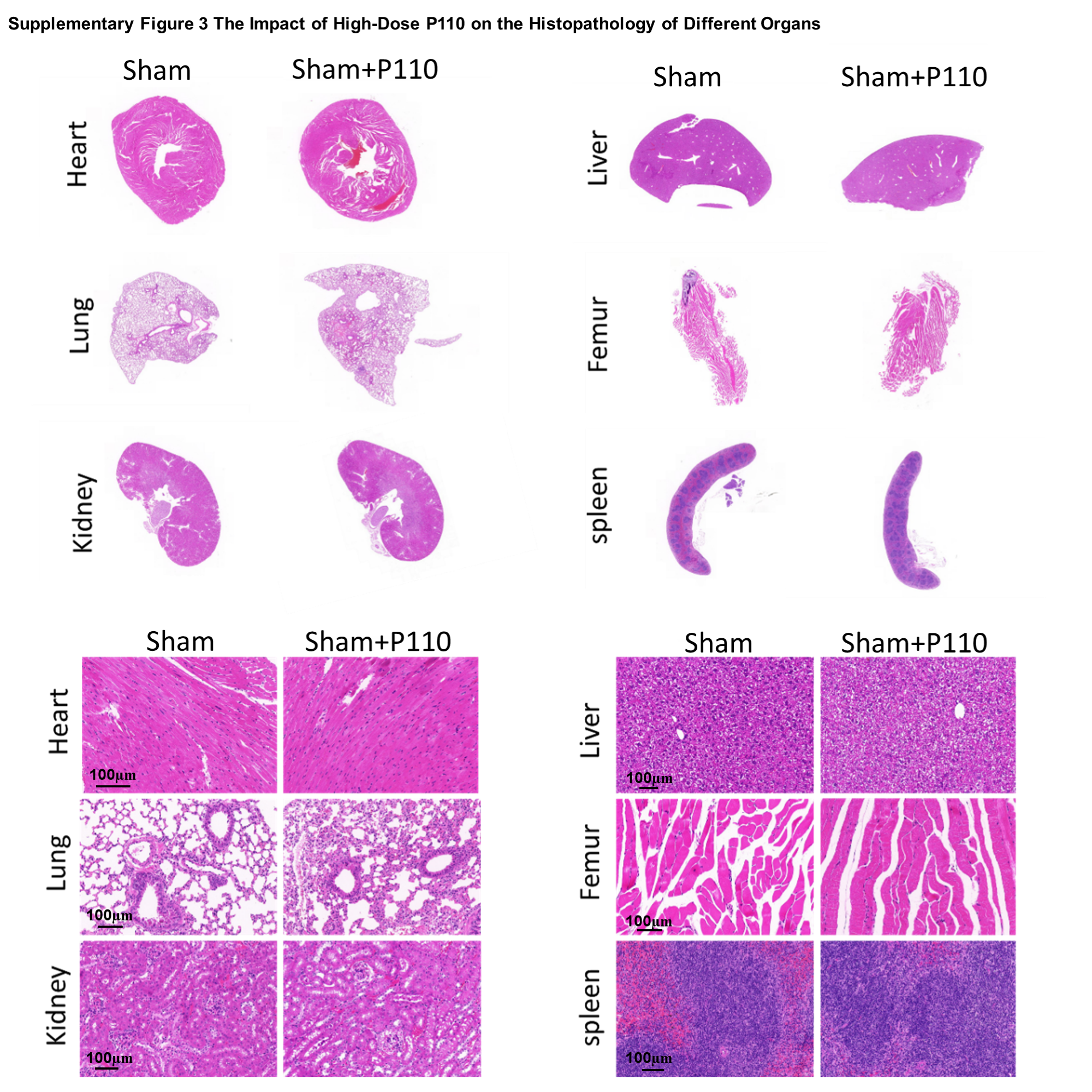

Supplement: Supplementary file 3 — Additional file 3: Figure S3. The Impact of High-Dose P110 on the Histopathology of Different Organs. A Representative images of H&E staining in heart、lung、kidney、liver、femur and spleen. B Representative images of H&E staining in heart、lung、kidney、liver、femur and spleen, Scale bar = 100 µm. [file 11658_2024_553_MOESM3_ESM.tif]

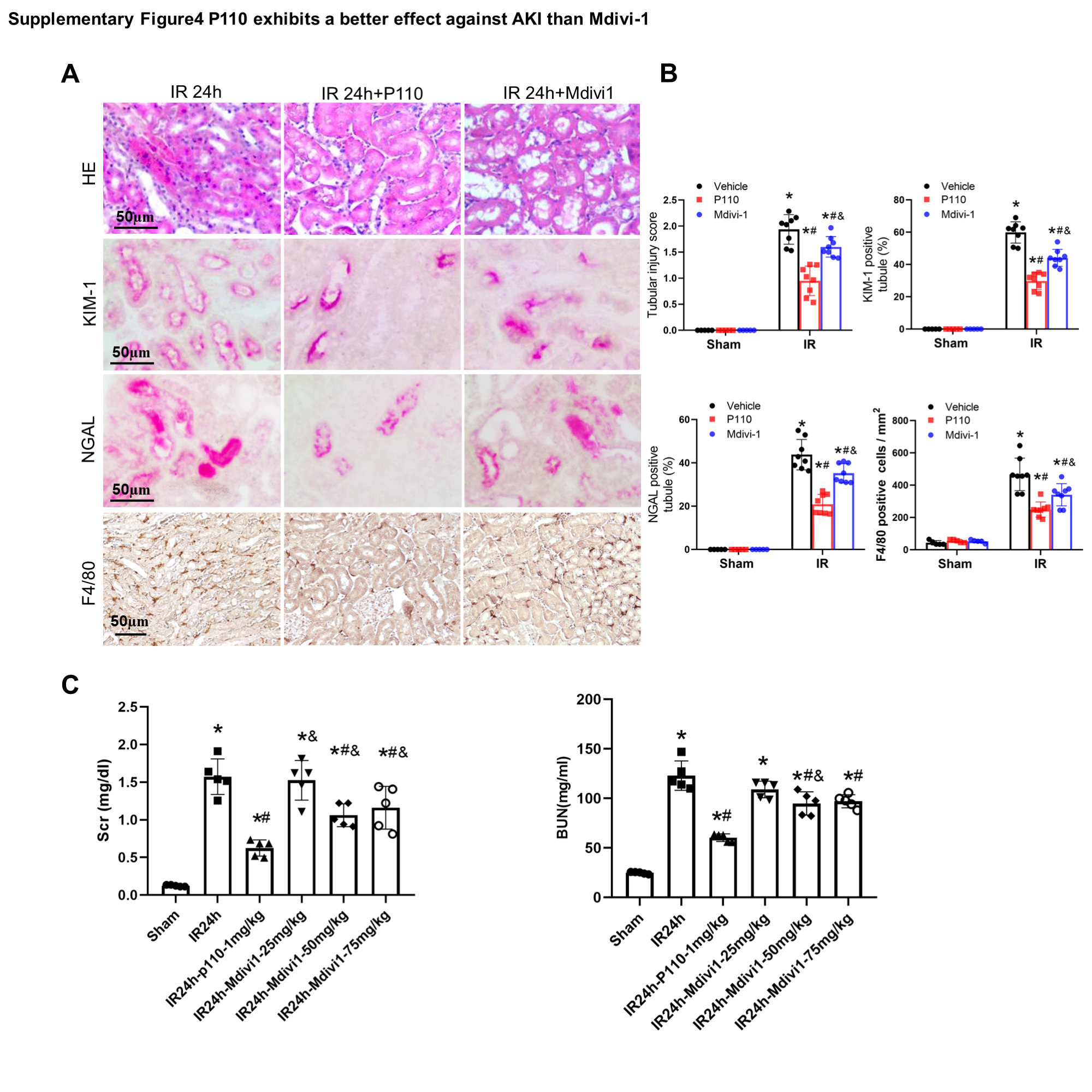

Supplement: Supplementary file 4 — Additional file 4: Figure S4. P110 exhibits a better effect against AKI than Mdivi-1. A Representative images of H&E staining. Scale bar = 50 µm. Pathological score of tubular damage. B Pathological score of tubular damage, Quantification of KIM-1,NGAL and F4/80 positive tubules. C The serum creatinine level and BUN level. Quantitative data are expressed as mean ± SD. *P < 0.05 versus respective Sham group. #P < 0.05 versus IRI + Vehicle group. &P < 0.05 versus IRI + P110 group. [file 11658_2024_553_MOESM4_ESM.tif]

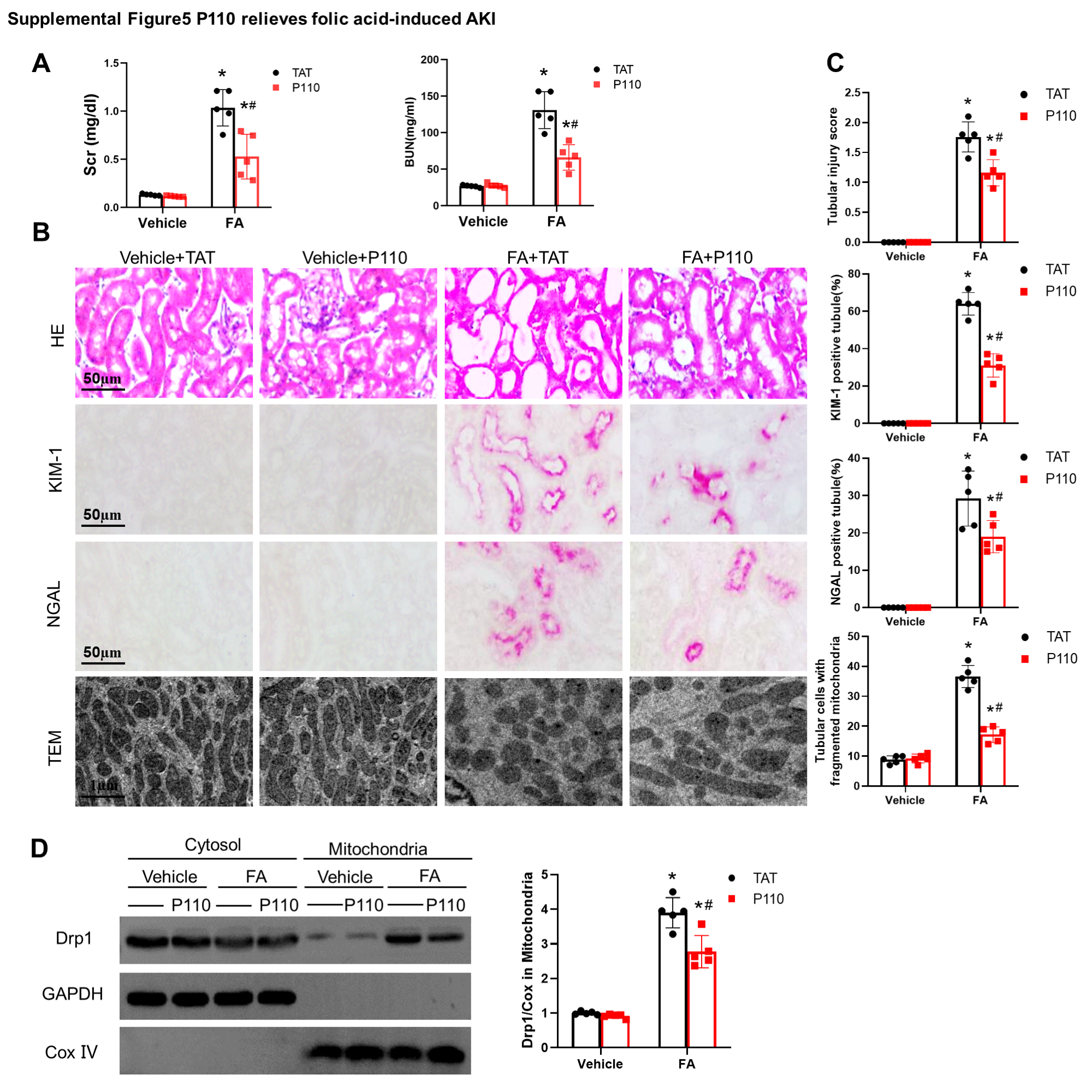

Supplement: Supplementary file 5 — Additional file 5: Figure S5. P110 relieves folic acid-induced AKI. A The serum creatinine level and BUN level. B Representative images of H&E staining,KIM-1 and NGAL immunohistochemistry and electron micrographs of mitochondrial morphology in proximal tubule cells. Scale bar = 50 μm. C Pathological score of tubular damage,Quantification of KIM-1 and NGAL positive tubules. D Representative Immunoblot and densitometry analysis of Drp1 in renal cytosolic and mitochondrial fractions. COX IV and glyceraldehyde-3-phosphate dehydrogenase (GAPDH) were used as loading controls of mitochondrial and cytosolic fractions, respectively. Quantitative data are expressed as mean ± SD. *P < 0.05 versus respective Sham group; #P < 0.05 versus FA group. [file 11658_2024_553_MOESM5_ESM.tif]

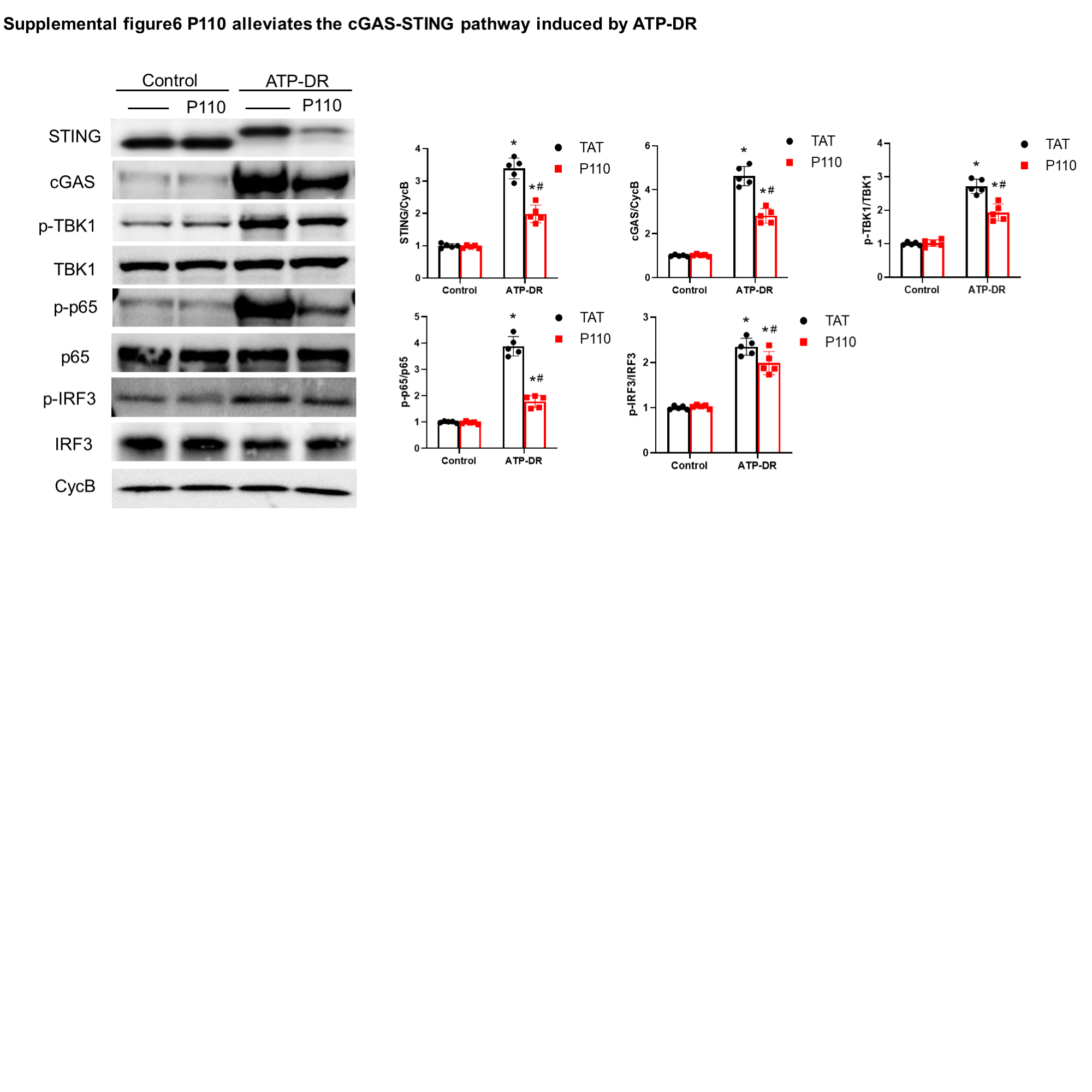

Supplement: Supplementary file 6 — Additional file 6: Figure S6. P110 alleviates the cGAS-STING pathway induced by ATP-DR. A, B Representative Immunoblot and quantitative analysis of STING,cGAS,p-TBK1,p-p65 and p-IRF3 in mPTC cells. Quantitative data represent the relative ratio to total TBK1 or p65 or IRF3.Cyclophilin B (CycB) was used as loading control. Quantitative data are expressed as mean ± SD. *P < 0.05 versus respective Control group; #P < 0.05 versus ATP-DR group. [file 11658_2024_553_MOESM6_ESM.tif]
