# Supplementary material for: Inhibition of Drp1- Fis1 interaction alleviates aberrant mitochondrial fragmentation and acute kidney injury
Source: Cell Mol Biol Lett. 2024 Mar 4;29:31. doi: 10.1186/s11658-024-00553-1 (PMC10910703; doi:10.1186/s11658-024-00553-1)
Supplement: Supplementary file 7 — Additional file 7. Primer sequences used in this study. [file 11658_2024_553_MOESM7_ESM.docx]

**Additional file 7: Table S1**

| Gene | Forward Primer | Reverse primer |
| --- | --- | --- |
| GAPDH | AAGTTCAACGGCACAGTCAA | TCTCGCTCCTGGAAGATGG |
| IL-1β | TCCTCTGTGACTCGTGGGAT | TGGAGAATACCACTTGTTGGCT |
| IL-6 | CTGCTCTGGTCTTCTGGAGTT | AAGTGCTGCTACCCTGAGATG |
| TNF-a | AGAACTCAGCGAGGACACCA | TCTGCCAGTTCCACATCTCG |
| Cgas | GTCGGAGTTCAAAGGTGTGGA | GACTCAGCGGATTTCCTCGTG |
| Irf7 | CAATTCAGGGGATCCAGTTG | AGCATTGCTGAGGCTCACTT |
| Ifit1 | CAAGGCAGGTTTCTGAGGAG | GACCTGGTCACCATCAGCAT |
| Ifit3 | TTCCCAGCAGCACAGAAAC | AAATTCCAGGTGAAATGGCA |
| MCP-1 | CTGAGTTGACTCCTACTGTGGA | TCTTCCCAGGGTCGATAAAGT |
| Isg15 | CTAGAGCTAGAGCCTGCAG | AGTTAGTCACGGACACCAG |
| Cxcl10 | CCAAGTGCTGCCGTCATTTTC | GCTCGCAGGGATGATTTCAA |
| Ifnb1 | CCCTATGGAGATGACGGAGA | CCCAGTGCTGGAGAAATTGT |
| Nampt | TACTGTGGCGGGAAT TGCTC | GCCGTTATGGTACTGTGCTCT |
| Pgc1a | GCTCTTCCTTTAACTCTCCGTGTC | CTTGACCTGGAATATGGTGATCGG |
| Sirt3 | AGGGGAAGACATATGGGCTGA | GGAGTAGGAACCTTGCAGGC |
